# Predicting human health from biofluid-based metabolomics using machine learning

**DOI:** 10.1038/s41598-020-74823-1

**Published:** 2020-10-19

**Authors:** Ethan D. Evans, Claire Duvallet, Nathaniel D. Chu, Michael K. Oberst, Michael A. Murphy, Isaac Rockafellow, David Sontag, Eric J. Alm

**Affiliations:** 1 Department of Biological Engineering, Massachusetts Institute of Technology, Cambridge, MA 02139 USA; 2 CSAIL, Massachusetts Institute of Technology, Cambridge, MA 02139 USA; 3 Present Address: Biobot Analytics, Somerville, MA 02143 USA; 4 Present Address: Superpedestrian, Cambridge, MA 02139 USA

**Keywords:** Machine learning, Diagnostic markers, Mass spectrometry, Diseases

## Abstract

Biofluid-based metabolomics has the potential to provide highly accurate, minimally invasive diagnostics. Metabolomics studies using mass spectrometry typically reduce the high-dimensional data to only a small number of statistically significant features, that are often chemically identified—where each feature corresponds to a mass-to-charge ratio, retention time, and intensity. This practice may remove a substantial amount of predictive signal. To test the utility of the complete feature set, we train machine learning models for health state-prediction in 35 human metabolomics studies, representing 148 individual data sets. Models trained with all features outperform those using only significant features and frequently provide high predictive performance across nine health state categories, despite disparate experimental and disease contexts. Using only non-significant features it is still often possible to train models and achieve high predictive performance, suggesting useful predictive signal. This work highlights the potential for health state diagnostics using all metabolomics features with data-driven analysis.

## Introduction

While fundamental to personalized healthcare, it is often challenging to diagnose an individual’s health state (a general term encompassing disease and non-disease phenotypes like age) due to low test sensitivity, specificity or the requirement of invasive procedures. Body-fluid sampling (e.g. blood or urine) offers a minimally invasive approach to identify health conditions throughout the body. The traditional concept of biofluid-based diagnostics relies on health-state biomarkers. Biomarkers cover a broad spectrum of measurements^1^, but typically refer to a small number of select and specific molecules or biopolymers, capable of differentiating healthy from diseased states. Currently, many biomarker-containing tests are used in routine lab monitoring (e.g. complete blood count, ‘basic’ and ‘comprehensive’ metabolic panels, lipid panels, etc.) providing coarse health-state categorization. Tests for many diseases exist and display a range of sensitivity and specificity, examples include: apolipoprotein E along with other measurements for Alzheimer’s disease^2^, the prostate-specific antigen test for prostate cancer^3^, alpha fetoprotein (AFP) for liver cancer^4^, as well as a recent use of the SOMAscan^5^ for diagnosing tuberculosis^6^.

Metabolomics rapidly supplies information on thousands of molecules, and provides a method for biofluid-based diagnostics^7–9^. To date, serum, plasma, urine and cerebrospinal fluid (CSF) metabolomics has been applied to many health states, ranging from cancers^10–13^ and infectious diseases^14,15^ to chronic obstructive pulmonary disease (COPD)^16^, smoking^17^ and Alzheimer’s disease^18,19^. Metabolomics studies are regularly performed using analytical instrumentation like liquid or gas chromatography mass spectrometry (LC–MS and GC–MS respectively) as well as nuclear magnetic resonance (NMR)^20^. Frequently, the goal is to determine the chemical identity of the features that are significantly altered between health states. For LC- and GC–MS studies, a feature is defined by a mass-to-charge ratio (mz), retention time (rt) and intensity. While a chemical name cannot be assigned to the majority of features, analysis of those that are identified allows for biological interpretation by differential analysis and biochemical pathway mapping^18,21^. Select chemically identified features are often used for differential diagnostics or health state association. For instance, certain amino acids have been associated with diabetes^22^ as well as urinary formate, alanine, and hippurate with blood pressure^23^.

For diagnostic modeling purposes, full metabolomics data sets are generally not used for training, validation, and testing. To deal with the large number of features, a host of feature selection methods and classification techniques are employed. Univariate statistical tests (Student’s t-test or Mann–Whitney U-test, MW-U) are routinely used to isolate statistically significant features—usually identified using false discovery rate (FDR) adjusted P-values < 0.05. Numerous other methods for feature selection are employed and cover multivariate statistical analysis, feature enrichment^24^, manual and statistical curation^25^, and the use of discovery cohorts to suggest metabolites for targeted analysis and model development^19,26^. Select feature sets are then used by algorithms like partial least squares discriminant analysis (PLS-DA) for both additional dimensionality reduction and health-state classification. Machine learning (ML) classifiers including random forests (RFs)^27–29^, support vector machines (SVMs)^30^ as well as neural networks^31^ and multiple forms of logistic regression^32,33^ have also been employed. Several ML classifiers have recently been compared on 10 curated data sets in a meta-analysis that found minimal, if any, improvement in classification when using SVMs, RFs or neural networks relative to PLS models^34^. Other analyses are strictly based on univariate or multivariate receiver operating characteristic-area under the curve (ROC-AUC)^29,35^.

We demonstrate across nine general health state categories that complete metabolomics data sets, combined with ML models, provide robust diagnostic performance. We performed standardized data processing and analysis on a set of 35 publicly available, predominantly untargeted LC- or GC–MS studies. In total, we analyzed 148 individual MS data sets covering diverse experimental conditions. Diagnostic classification was performed using a simple and interpretable ML model: logistic regression with L1 regularization (L1-LR). This regularization technique inherently selects the subset of features to perform diagnosis, reducing dimensionality without requiring statistical significance calculations. Models trained on all features outperformed those that used only statistically significant features. Moreover, models often achieved high classification performance using only non-significant features. These results were observed across all biofluid types and analytical methods, with the major determinant of performance being the disease or health state analyzed. Of the nine health state categories, cancer was the most challenging to diagnose and displayed the largest range of model performance. In contrast, health states including infectious diseases, cardiovascular, and rheumatologic states, showed high predictive performance. These results may suggest that biofluid-based metabolomics is a promising technology for health state diagnostics.

## Results

### Biofluid-based metabolomics studies cover many health states with various cohort sizes

**Figure 1:**
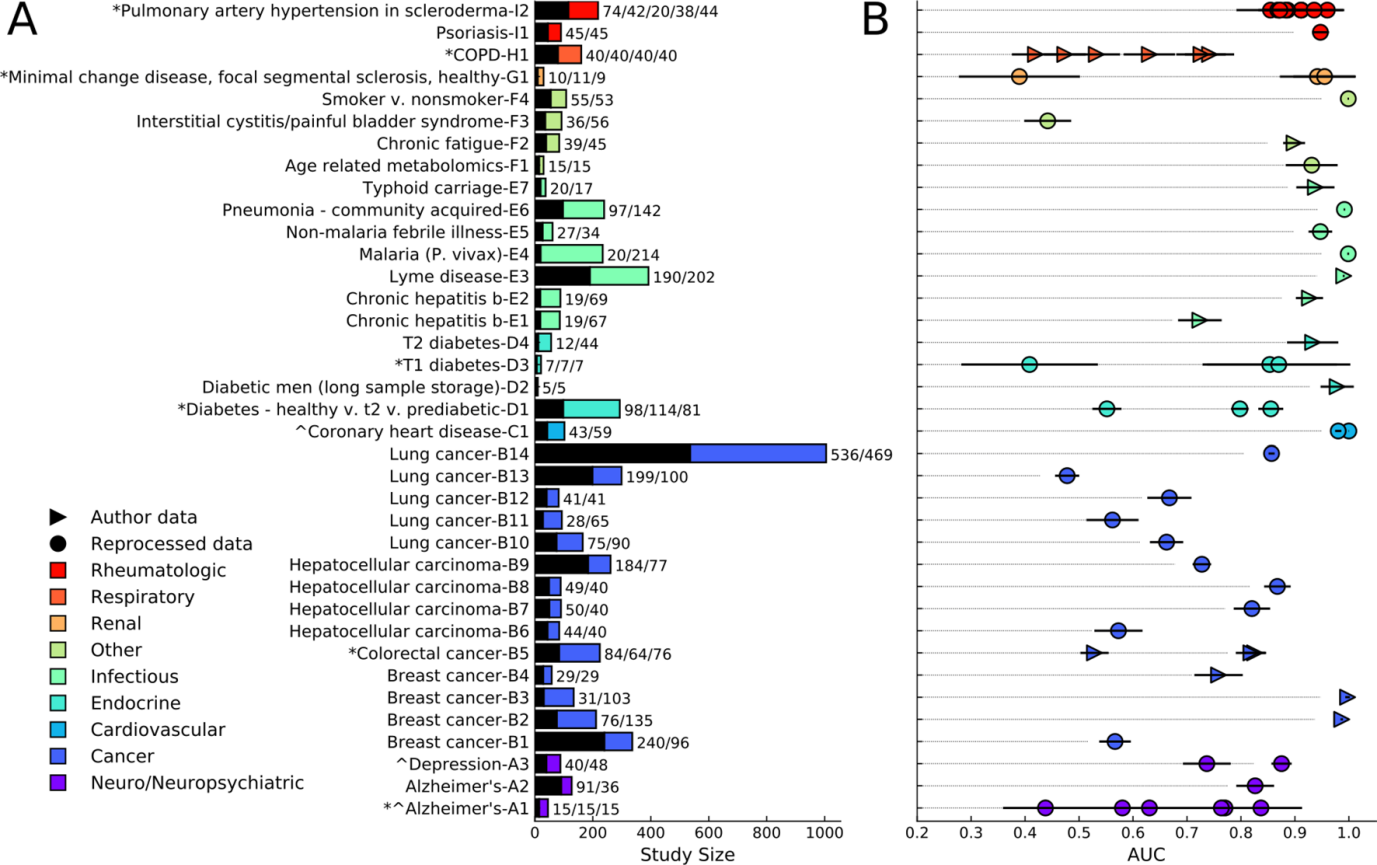
Body fluid based metabolomics often possesses health state-dependent signal and diagnostic capability. (**A**) Studies analyzed and their associated cohort sizes (control/case sizes to the right of the bar plot), separated by health state category. Shown in black are the controls, with the cases in color. Multiclass control bars correspond to the size of the first class for 3-class studies, or first 2 classes of 4- or 5-class studies, with the case bar representing the remaining samples. Multiclass studies are: D1 (Type 2 diabetes, prediabetic, healthy), H1 (never smokers, former smokers, smokers, COPD patients), D3 (Type 1 diabetes—insulin injection, Type 1 diabetes—insulin withdraw, no diabetes), A1 (Alzheimer's disease, mild cognitive impairment, normal), B5 (colorectal cancer patients, polyp patients, healthy controls), G1 (minimal change disease, focal segmental glomerulosclerosis, control), I2 (normal, pulmonary artery hypertension, low risk, healthy, borderline pressure). (**B**) Averaged ROC-AUC and standard deviation analysis for 30 L1-LR models, each trained and tested on different randomized, stratified shuffles of the within-study combined data sets. *Multiclass studies for which one-vs-one models were built. ^Studies for which it was not possible to combine data sets.

Literature and database searches identified 35 studies, covering nine general health state categories (Fig. 1A). While one lung cancer study^28^ provided data for 1005 individuals, most studies included tens to a few hundred individuals. Four types of cancer represented the largest category, with 14 studies^11,12,24,26,28,36–43^; cardiovascular^35^, rheumatologic^29^^§^, renal^§^, and respiratory diseases^16^ were represented by only one or two studies. Other health states with a small to medium number of studies included neuro/neuropsychiatric^18,19^^§^, endocrine^21,27,44,45^, and infectious disease^14,15,25,30,46,47^, as well as a general ‘other’ category^17,48–50^.

We reduced complex studies to single or multiple binary classification problems, typically between a control state and disease or altered health state. As an example, Alzheimer’s study A2^19^ had an intricate discovery cohort and targeted validation set that looked at differences between healthy controls, individuals before and after conversion to a cognitively impaired state, and individuals who entered the study with cognitive impairment. For this study, we analyzed the untargeted mass spectrometry (MS) data, which included only healthy and pre-study cognitively impaired individuals. For one breast cancer study^42^, we analyzed the serum (B2) and plasma (B3) data separately, as the study included differently sized discovery and validation cohorts with different sample types. The seven multiclass studies were analyzed via multiple one-to-one comparisons as done in many metabolomics studies (Fig. 1A). The chronic hepatitis B study^15^ was split in two because the oxylipin assay (E1) possessed a different number of cases from the lipid analysis (E2). One breast cancer study^43^ (B1, Table S1) aimed to generate prognostic predictions for response to chemotherapy among patients with tumors.

### Biofluid profiles enable health state prediction using a data-driven approach

Metabolomics features from LC- and GC–MS studies across the nine health states generally provided moderate (0.7–0.9 AUC) to high (> 0.9 AUC) predictive performance (Fig. 1B). When raw MS data was available, we reprocessed it using an in-house pipeline, generating feature tables of peak intensities associated with each mz-rt pair (referred to as ‘reprocessed’ data sets, *Methods*). If only preprocessed feature tables were available, all features were used (referred to as ‘author’ data sets); the majority of author data sets consisted of all features or named metabolites (B2–5, D2, D4, E1, E2, E7, F2, H1), study E3 provided features with statistical associations with the outcome of interest. To minimally bias our conclusions and treat all data in a similar manner, each data set was percentile normalized^51^. For each study with multiple data sets, we combined all data into a single data set (e.g. concatenating the positive and negative ionization mode data from an LC–MS study) when possible; for three studies^18,35^^§^ it was not possible to match samples across data sets (Fig. 1A). To establish baseline predictive performance, we trained L1-regularized logistic regression (L1-LR) models for each study (Methods). Many displayed test AUC values > 0.7 for at least one comparison (Fig. 1B). For most multiclass studies, we typically observed either near random guessing (AUC ~ 0.5) or AUC values greater than 0.7, depending on which one-versus-one health state comparison was performed. Nearly all of the infectious disease models displayed significant predictive power, even for studies with small cohorts. Rheumatologic, cardiovascular, neuro/neuropsychiatric, and select ‘other’ health states possessed similarly high AUC values. In contrast, seven out of 14 cancer studies displayed low model performance (< 0.7), and the interstitial cystitis/painful bladder syndrome study yielded random model guessing.

### Individual data sets from different experimental conditions display mixed predictive performance

**Figure 2:**
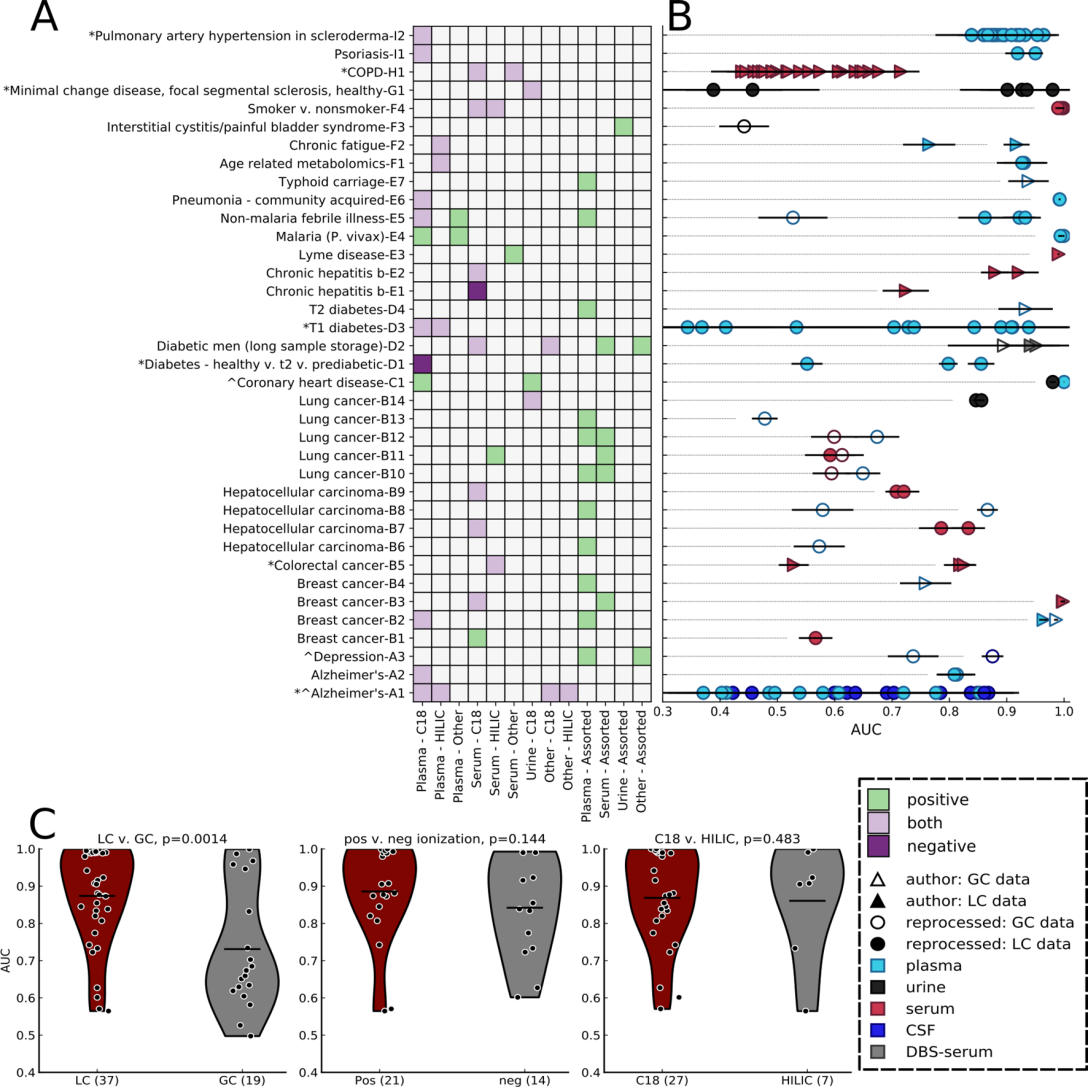
Health state information is found in all body fluids using different instruments, mass spectrometry ion modes, and chromatographic methods. (**A**) Ion mode, sample, and column type (for liquid chromatography) used for each individual data set. (**B**) Individual data set ROC-AUC and standard deviation analysis of 30 averaged L1-LR models, each trained and tested on different randomized, stratified shuffles, with associated sample and instrument type. (**C**) Violin plots for the comparison of non-multiclass, liquid chromatography to gas chromatography data sets (left), positive versus negative ion mode for all liquid chromatography data sets (middle), and C18 versus hydrophilic interaction chromatography (HILIC) columns (right), using AUC values from models trained on individual data sets, using all features.

Models trained on individual data sets within a study generally performed well across different experimental conditions, including biofluid type, method of chromatographic separation, MS instrumentation, and ionization mode. Plasma and serum accounted for the vast majority of studies, with only four using urine, two using CSF, and one using dried blood spots (DBS, Fig. 2A). We found that sample type did not significantly affect model performance, with AUC values predominantly reflecting the disease or health state under study. Likewise, plasma- and serum-based studies displayed no statistically significant difference in test set AUCs (Fig. 2B and Figure S1). For the chronic hepatitis B study, models built with the positive or negative ionization mode lipidomics data (E2) substantially outperformed the oxylipin assay data (E1, Fig. 2B). When analyzing studies with only two classes, LC (including both hydrophilic interaction chromatography (HILIC) and reverse phase C18 chromatography and its variants) outperformed GC (P = 0.0014 MW-U test, Fig. 2C). However, a disproportionate number of GC data sets (12 of 19 as opposed to 10 of 37 for LC) were from cancer studies, the most challenging diagnostic category. Further, LC-based studies generally possessed 1–2 orders of magnitude more features for model training (Figure S2). Most studies used either both or only positive MS ionization mode. Two used solely negative mode and each displayed test AUCs > 0.7 for at least one comparison. For binary class LC–MS data sets, ionization mode and column type did not appear to alter predictive performance (Fig. 2C). These results may be biased by relatively small sample sizes in addition to study- or health state-specific experimental parameters.

### Biological considerations may explain select low performance models

Several low performing models originated from multiclass studies—specifically, comparisons between similarly presenting health states. Low AUC values were seen in studies on Type 1 diabetes, Type 2 diabetes, Alzheimer’s disease, colorectal cancer, COPD, and one study on two nephrotic syndromes: minimal change disease (MCD, a kidney disease characterized by significant urine protein levels) versus focal segmental glomerulosclerosis (FSGS, scarring of the kidney that may also present with high protein levels in urine, Figure S3). For instance, in the colorectal cancer study, it was not possible to distinguish between healthy individuals with non-cancerous polyps versus those without (AUC = 0.529 ± 0.009, mean and 95% confidence interval respectively). However, we were able to differentiate true cancer cases from both healthy states (AUCs = 0.819 ± 0.010, 0.825 ± 0.007). Similarly, differentiating between MCD and FSGS was not possible (AUCs = 0.388 ± 0.136 and 0.457 ± 0.132 for positive and negative ionization LC–MS, respectively), yet both were easily distinguished from healthy controls (AUCs > 0.9). This pattern extended to other health states with multi-class studies (Figure S3). These cases highlight the difficulty of differentiating health states with similar metabolic signals, something that appears increasingly common as the number of classes increases.

### Using all features, not solely statistically significant features, provides the best performance

**Figure 3:**
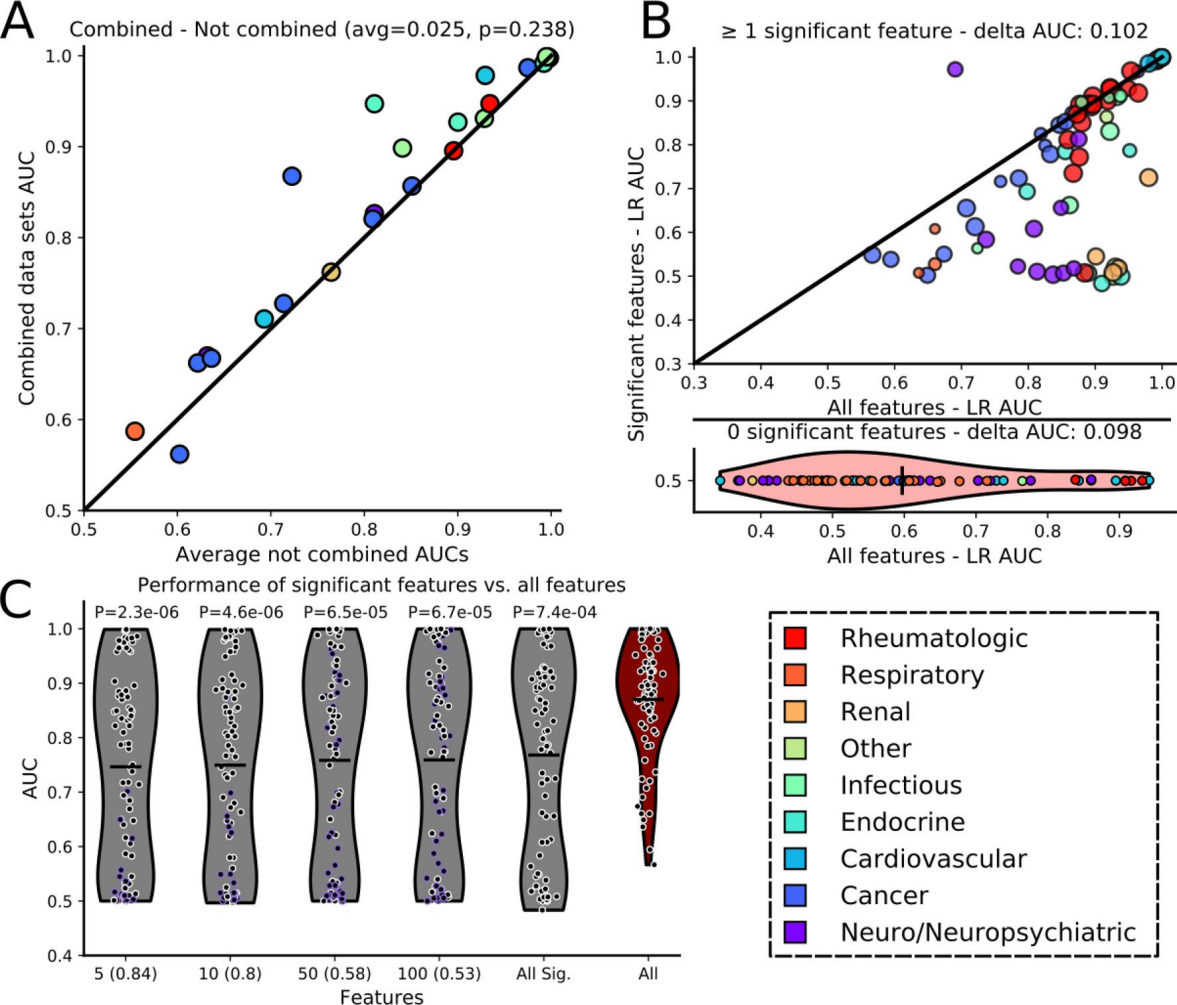
Using all features generally leads to the best model performance. (**A**) Comparison of AUC values from L1-LR models built from within-study combined data sets versus the average AUC of independent models built on non-combined data sets. The average difference between combined and non-combined model AUCs, along with the P-value for the comparison (MW-U test). (**B**, top) Comparison of model performance on individual data sets with at least one statistically significant feature. Circle size is proportional to the number of features. The purple outlier point with better AUC using only significant features is from a HILIC, CSF sample in positive ionization from study A1. (**B**, bottom with shared y-axis label) Comparison of performance between models trained using all features versus ‘significant feature only’ models when there were no significant features with accompanying violin plot of the AUC distribution of models built using all features. Delta AUC values were determined by subtracting the AUC of a model built with only significant features from that of a model trained on all features, and then averaged over all data sets. (**C**) Comparison of models built using up to 5, 10, 50 and 100 of the most significant features (lowest Q-value) or all significant features, versus models trained using all features; results displayed only for data sets that possessed significant features. Purple outlines indicate data sets with fewer than the cutoff number of significant features and the fraction at the bottom depicts the number of data sets meeting the cutoff. P-values correspond to MW-U tests between the AUC values from models using all features versus the AUC values from models built with a select number of significant features. Health state color legend for (**A**) and (**B**) shown in the bottom right.

We next assessed the performance of L1-LR models using two separate data set modifications. The first tested whether there is more predictive power when using data combined from different experimental conditions (as done for Fig. 1B). We observed that the combined data set models generally performed on par with the average of models built using individual data sets (average AUC increase of 0.025, not statistically significant P = 0.238), with few combined data set models showing increased performance (Fig. 3A). This result illustrates that larger feature sets, putatively with more molecular information, may not lead to improved diagnostic performance. The minimal increase in performance further suggests that data from different experimental conditions contains redundant information. For this reason, we chose to focus our analysis on models trained on individual data sets.

The second modification tested the effect of reducing the feature space to only statistically significant features in a manner similar to how most studies identified such features (FDR corrected P-values < 0.05). We split data sets into two groups: those with at least one significant feature during training, and those without (study E3 was removed). For data sets with significant features, 75% of models trained using all features outperformed those that used only statistically significant features (Fig. 3B, top). For the other group, lacking features to train a model with, the data sets were given an AUC of 0.5, representing random guessing (Fig. 3B and Methods). In comparison to these random models, models trained with all features displayed a ~ 0.1 average increase in AUC unless overtrained (Fig. 3B, bottom).

While using all features appeared optimal, significant features alone did show predictive power. Thus to study their importance and information content we tested model performance on subsets of the most significant features. Using up to 5, 10, 50 or 100 (or all in cases where the number of features was less than one of these values) of the most significant features demonstrated that—for each comparison—the models built with all features outperformed the significant feature-based models (Fig. 3C). This analysis excluded the 0.5 AUC-assigned data sets that lacked significant features. However, when these data sets were retained, the difference in model performance remained (Figure S4). For most data sets, using any number of significant features resulted in similar predictive power (Figure S5); demonstrating that a small number of significant features accounted for the majority of predictive power of the significant features.

### Machine learning model comparison

Observing improved performance in models trained using all features, we tested whether the class of model affected performance. To first ground our study with a commonly used model, we compared the performance of L1-LR models to partial least square discriminant analysis (PLS-DA) models, with both model types using all the available features. The two models performed similarly in terms of AUC, accuracy, specificity, sensitivity, precision and Matthews correlation coefficient (Data S1). However, the L1-LR models may offer increased interpretability as they provide sparse sets of explanatory features (Figure S6). We further compared the L1-LR models with four other models: K-nearest neighbors (KNN), naïve Bayes (NB), support vector machines (SVM) and random forests (RF). This comparison showed the L1-LR model to perform better than both KNN and NB models and similarly to SVMs and RFs (Figure S7). The RF models had a slightly higher average AUC (difference = 0.03). Much of the improvement originated from study (I2) that included 20 individual data set comparisons. All of the 20 data sets were of medium sample size (~ 60–120) but possessed a very large number of features (~ 29,000), which may have resulted in overfitting (study D3 displayed a similar outcome with many features and a much smaller study size, Figure S8).

### L1-LR provides sparse models that use both statistically significant and non-significant features

**Figure 4:**
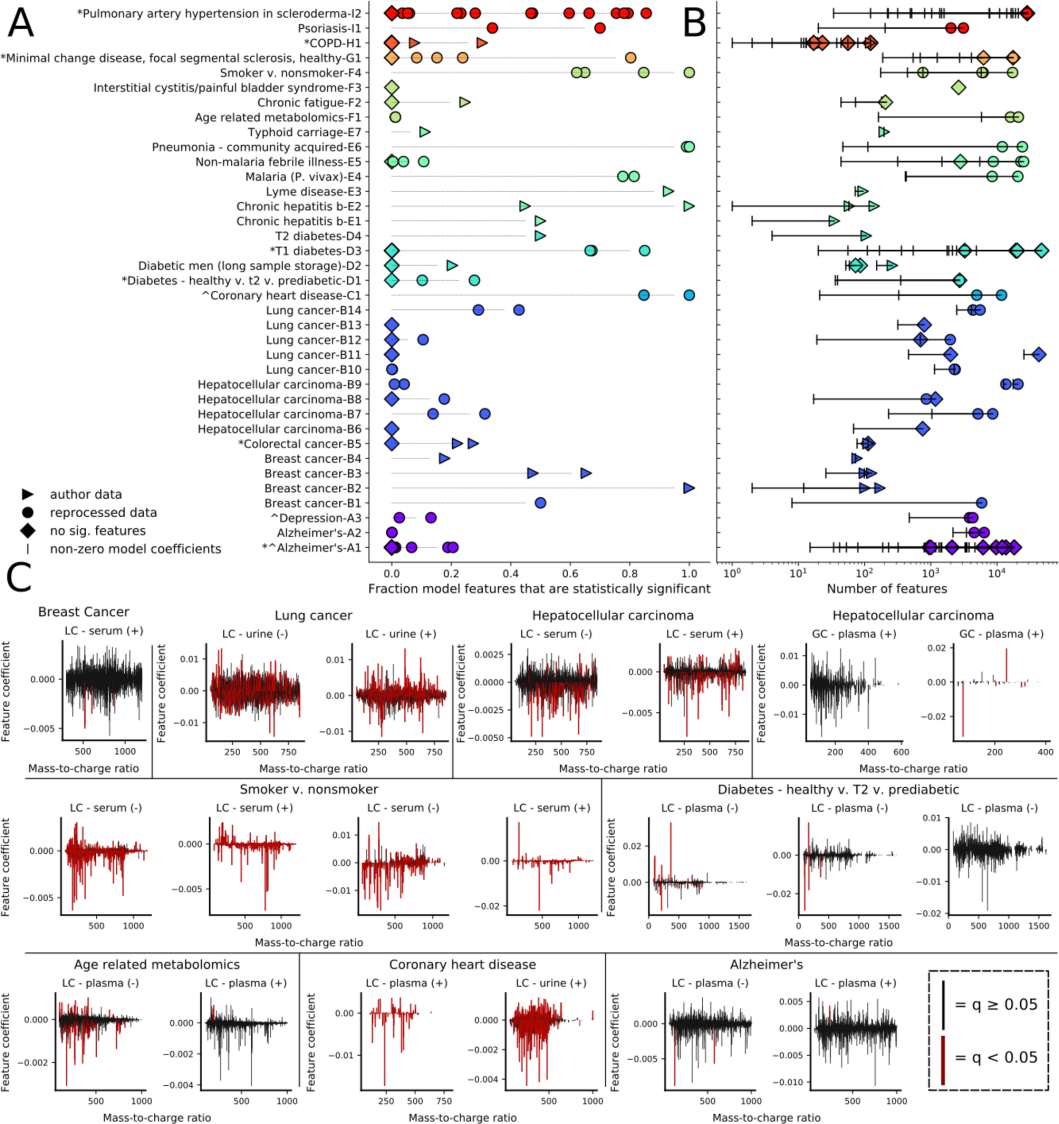
Machine learning models are relatively sparse and frequently use features spanning a large mass range and both significance types. (**A)** Fraction of non-zero feature coefficients in the models corresponding to statistically significant features (p < 0.05 FDR-corrected, MW-U test) for single models trained on individual data sets. (**B**) Number of input features (colored points) relative to the number of non-zero model features (vertical dashes) for a single, representative model training for each data set. (**C**) Representative plots of the features and associated average model coefficients (30 model trainings) used across the range of observed mass-to-charge ratios. Significant versus non-significant features are depicted in different colors (red, q < 0.05; black, q ≥ 0.05). Data sets (clockwise, starting from the upper left): breast cancer (B1), lung cancer (B14), hepatocellular carcinoma (B7), hepatocellular carcinoma (B8), diabetes (D1), Alzheimer’s disease (A2), coronary heart disease (C1), age (F1), smoking versus non-smoking (F4).

We examined the extent to which L1-LR models trained on all features across different health states recovered significant features, as well as the degree to which the features that were used (i.e. had non-zero coefficients) were non-significant. We found that the models used a wide range of statistically significant features, from zero to almost all (Fig. 4A). While this affirmed the importance of significant features, for many models, non-significant features constituted a large fraction of the features used. In fact, among models trained on data sets with at least one significant feature, AUC only showed partial correlation (R = 0.54) with the fraction of significant features used (Figure S9).

Relative to the number of input features, L1-LR models trained using all features provided sparse solutions, supplying increased interpretability by implicit feature selection (Fig. 4B). Analyzing a single model for each data set, we found that a large range of total features were used (from tens to tens of thousands); but that the number used was generally reduced by an order of magnitude relative to the number of input features (Fig. 4B, vertical dashes). This reduced feature space may identify features, notably those with high model coefficients, that are especially important for a given predictive task and follow up analysis. Chemical identification of these features would allow for biochemical and systems-level analysis to better understand these diseases or health states.

### Features with a diverse set of properties are used

Trained models used a relatively large number of features with small coefficients (< 0.005), spanning a range of mz and rt values (Fig. 4C and Figure S10). Only a handful of features possessed relatively large model coefficients (> 0.005). Select models primarily used significant features (F4), while others used mostly non-significant features or a mixture of both (B1); importantly, in both cases features coefficients could be large or small for either significance type (Fig. 4C). Greater model coefficients were often observed for features with larger enrichment factors between cases and controls; however, a majority of features possessed near zero feature coefficients, limiting analysis (Figure S11). Across studies, models used features from the majority of the mz domain (from ~ 50 to > 1,000 Daltons). This observation putatively suggests that many different molecules in biofluids may provide health state information.

### Non-significant features alone can provide high model performance

**Figure 5:**
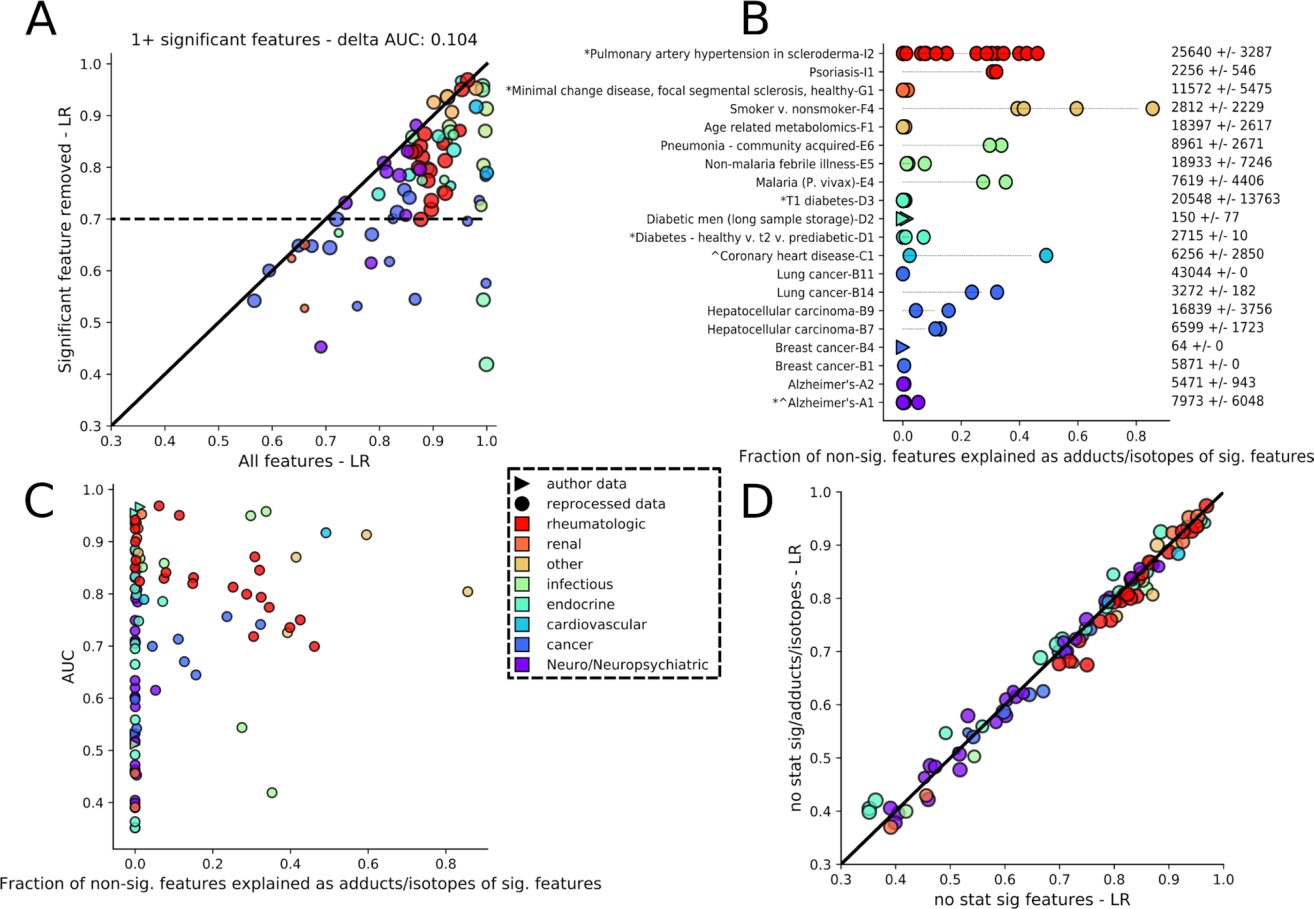
Models trained with only non-significant features often retain relatively high diagnostic performance. (**A**) AUC comparison between models trained using all features and those using no statistically significant features for data sets with at least one significant feature. Circle size is proportional to the log of the number of features in the data set. (**B**) Fraction of non-significant features for each high resolution mass spectrometry data set that can be explained as adducts or isotopes of statistically significant features. Numbers on the right are the average number of features across a study’s data sets, with standard deviation. (**C**) AUC of models trained and tested using only non-significant features versus the fraction of non-significant features explained by adducts and isotopes in the input data set. (**D**) AUC comparison between models trained using only non-significant features versus non-significant features without adducts or isotopes of significant features. Circle size is proportional to the log of the number of features in data sets from which significant features, their adducts, and isotopes have been removed.

In light of the improvement observed using all features, we investigated the predictive capabilities of only the non-significant features. We removed all significant features from each data set (and study E3) and trained models on the remaining non-significant features. A surprising number (73%) of models still achieved high AUC values of ≥ 0.7 (Fig. 5A, dashed line). As a reference, 90% of models trained using all features (for these data sets) achieved an AUC ≥ 0.7, with an average AUC difference of 0.104 between the two cases. Additionally, we observed high AUC values across multiple health state categories and experimental parameters (Fig. 5A,B).

We verified that the performance was not due to information from the significant features remaining in their non-significant isotopes and adducts. For this, we analyzed the high resolution (HR) MS data sets in which isotopes and adducts could be determined. In nine of the 20 HR-MS data sets, more than 10% of the non-significant features could be explained as putative adducts or isotopes of the significant features (Fig. 5B, Methods). Furthermore, all nine displayed AUC values > 0.7. Among data sets with a large number of total features, some had a high representation of isotopes and adducts while others had relatively few (Fig. 5B). However, low numbers of isotopes and adducts likely arose due to data sets possessing only a small number of significant features (Figure S12). Additionally, data sets with a larger number of significant features tended to come from studies with larger cohorts as opposed to simply having the most initial features (circle sizes, Figure S12). Studies with and without a large fraction of putative isotopes and adducts of significant features displayed a range of AUC values, giving initial support to them playing a minimal role in classification (Fig. 5C). After removing significant feature isotopes and adducts, newly trained models showed a linear relationship with those that included isotopes and adducts (Fig. 5D). The features used by these models spanned a similar range of MS intensity values to those used in previous models and were not biased to background (low intensity) features (Figure S13). A similarly wide mz-range of features possessed useful predictive information, despite being non-significant (Figure S14). Like full data set-trained models, a handful of features possessed large absolute coefficient values (0.01–0.4) while many had very small coefficient features (< 0.0005). While this analysis did not account for possible in-source fragmentation by-products, given the extent of features used, across most of the mz-space, it is likely the high AUC values were not simply a result of remnant information from the significant features and point to a route to uncover important features.

## Discussion

This analysis evaluated the predictive power of biofluid metabolomics for machine learning based diagnostics. In many cases, biofluids provide robust diagnostic capabilities. Here we discuss: (1) the utility of all features along with the information content in metabolomics data, (2) health states suited for metabolomics diagnostics and those that are not, and (3) the challenge of cross-study comparison due to the host of experimental conditions and individual study goals. In light of the difficulty of cross-study comparison, we highlight limitations to the observed robustness of these results, and support efforts for standardization and data sharing.

Biofluid-based metabolomics provides rich diagnostic information, much of which is often overlooked—specifically, the non-significant features. As opposed to building models solely from significant features, we found that performing L1 regularization with complete feature sets yielded improved model performance. Moreover, even non-significant features were capable of providing, in select cases, robust health state discrimination. An additional benefit of the L1-LR model is its relative interpretability, as model coefficients may help identify important molecular features that could focus future research. As a note of caution, with possibly tens of thousands of features, overfitting is a major concern and may reveal many features that appear predictive but in reality are not (and might not even be biologically relevant). This may lead to higher performance when training with all features than with only statistically significant features. Further, for data sets with imbalanced class representation, real features that are not missing at random (e.g. smoking or other lifestyle choices) may have led to improved model performance; our requirement of a feature appearing in > 5% of samples may have removed such important features, resulting in improved performance by the unidentified, non-significant features. As such, verifying predictive models in separate cohorts is critical.

While mass spectrometry-based metabolomics displays substantial diagnostic information, for this purpose, it may possess a high level of information redundancy. Information redundancy is an explanation for why models trained via a combination of all within-study data sets did not substantially increase model performance relative to the average of models built on individual data sets (Fig. 3A). This hypothesis may also be an explanation for the minimal change in performance of models trained on the 5–100 most significant features relative to using all significant features. This redundancy may occur for several reasons. At the instrumental level, it may arise due to the high dimensionality of the data relative to the number of blood or urine metabolites (e.g. ~ 4000 in serum^52^ or ~ 2700 in urine^53^). The high-resolution mass spectrometry data may possess many isotope and adduct peaks that putatively hold the same information as the primary monoisotopic species. At the biological level, metabolites connected via biochemical pathways may provide information on each other. Additionally, the features may reflect a shared underlying state, despite different physicochemical properties. Thus, while the data sets come from different ionization modes, chromatography methods, or sample types, they may not possess orthogonal diagnostic information.

Our analysis found multiple health states suited for biofluid-based diagnostics along with others that are challenging to diagnose. High model performance across infectious diseases suggests this category may be an attractive area for diagnostics development. This high performance may have a biological explanation, as infection involves immune response carried via the circulatory system. A similar argument could be made for renal, cardiovascular and rheumatological health states that also display high model AUCs and involve the circulatory system. Of equal importance are challenging to diagnose health states (e.g. cancer), for which the wide range of predictive performance may stem from multiple issues. Some health states—especially in their early stages—may not be reflected in biofluid metabolite levels; in the case of cancer, this may occur due to immune suppression^54^. Individual responses to a given cancer (or other disorder) may be highly variable, and even tumor region specific^55^, possibly obfuscating any chemical signal in a biofluid. Large and diverse cohorts may minimize such variability—perhaps one reason why the large, 1005-person cancer study^28^ performed so well. Finally, predicting long-term neoadjuvant chemotherapeutic response using baseline metabolomics appears challenging (AUC ~ 0.5, study B1^43^) and may require additional information including electronic health records combined with other aspects of clinical machine learning^56^.

Determining which health states are unsuitable for metabolomics-based diagnostics on the basis of these results is difficult. Our goal was not to optimize individual model performance by engineering processing parameters, using optimal data set-specific transformations, or matching machine learning models to individual problems. Instead, we attempted to treat each data set as similarly as possible in order to comment more broadly on the potential of metabolomics for diagnostics. This may have led to certain data sets displaying lower than expected performance relative to reported values. For example, cancer study B5^11^ obtained high AUC values of 0.95 and 0.93, compared to our 0.82 ± 0.01 and 0.83 ± 0.01, for distinguishing colorectal cancer from non-cancerous polyps or healthy controls, respectively. Given these considerations, it is likely too early to rule out health states for which such diagnostics can be built. In particular, due to the diverse nature of cancers and because most studies used relatively small cohorts for either diagnostics or treatment response, this multifaceted disease may still be accurately characterized with metabolomics.

The studies we analyzed encompassed several distinct sample types, analyzed by many chromatographic and MS techniques, making cross-study chemical or biological comparisons difficult. Given these challenges within a single health state (e.g. cancer), it was not possible to make comparisons across health states. Such comparisons are necessary to validate the predictive profiles or features uncovered. As a result, it is unclear whether the metabolic profiles observed are health state-specific or simply general signals of illness. Thus, while it is ideal to match the proper experimental and analytical methods to the problem of interest, doing so makes cross-study comparisons more challenging and lends minimal insight for new health states for which these parameters are not known. It appears prudent to use all available routes of data collection when possible, despite the fact that different methods may generate data with overlapping information.

While GC–MS performed worse than LC–MS, this may not reflect the true capabilities of the method. Our data processing pipeline may be better suited for LC data as IPO^57^ (Isotopologue Parameter Optimization) was built for extracting parameters from LC data. A larger fraction of GC–MS studies were on cancer (63%), versus 27% of LC–MS studies. Additionally, more LC studies used HR-MS, possibly supplying more information. In fact, GC–MS is more amenable to between-lab comparisons^58^ and may supply information on a different set of molecules that are critical for diagnosis.

Considering the diversity of cohorts and the possible effects of confounding variables, it is challenging to ascertain the robustness of the diagnostic capabilities obtained. Many studies directly accounted for variable like age and sex in their cohort design^16,17,21,28,36–38,49^. This information was, however, not always available and to treat all studies equally, such variables were not directly modeled. Importantly, study-specific variables like medication, familial or genetic linkage, and lifestyle may impact the results obtained. Without access to detailed individual data, it is difficult to determine what metabolomics information is truly clinically relevant for diagnostics. This likely would limit the ability to transfer the results from one study to another. Furthermore, a number of the studies possessed small cohorts (< 30 individuals), thus the diagnostic signatures obtained may not translate to larger and more diverse populations. Measurement and inclusion of additional types of data would significantly boost the ability to learn across studies and generalize the results.

Many of the presented caveats support the metabolomics community’s efforts to standardize methods across labs and studies^59,60^, and underscore the importance of open-access sharing of data. Releasing raw MS data sets—accompanied by quality control and standard samples, run order, batch numbers, and secondary MS data—could facilitate improved data correction, compound identification, and comparisons between studies. The inclusion of general and health state-specific metadata, when permitted, would also be highly beneficial. This data would significantly advance the community’s ability to build off one another’s work and would expand the diagnostic capabilities of metabolomics to more challenging problems.

## Methods

### Study data acquisition

^§^Refers to studies without a published accompanying manuscript. This includes the following studies: ST000329, ST000763, ST00062/3. Studies were obtained from The Metabolomics Workbench, https://www.metabolomicsworkbench.org/ or Metabolights (https://www.ebi.ac.uk/metabolights/)^61^ with the exception of the study by Feng et al*.* that was obtained from https://datadryad.org/resource/doi:10.5061/dryad.s8k81 with additional data from their supplementary information. A full table listing the study identifiers, project IDs, and project DOIs is listed in Table S1. ST000062 was obtained directly from the study authors.

### Data processing

Python 3.6.5 with scikit-learn version 0.19.1 and R 3.5.1 were used. Following data acquisition, LC- or GC–MS files were converted to .mzML or .CDF using msconvert_ee.py (if needed), a python wrapper for msconvert in ProteoWizard^62^. Select .PEG and Agilent ChemStation .D files were converted to .CDF using a wrapper (PEG_to_CDF.py or chemstation_d_to_CDF.py) of Unichrom’s ucc.exe (https://www.unichrom.com/dle.php); conversion scripts were run on a Windows operating system.

Data was then feature extracted using full_ipo_xcms.py. This converted any .CDF files to .mzData format (using cdf_to_mzData.R), selected XCMS^63^ parameters (bw, peakMin, peakMax, ppm, noise, mzdiff, binSizeObi, gapInit, gapExtend, binSizeDensity) by averaging the results from IPO (using three independent outputs from IPO_param_picking.R, each run on a single random LC- or GC–MS file from all processable files), and finally extracted data set features (extract_features_xcms3.R with data set-specific command line flags determined via IPO; other XCMS parameters were hardcoded, e.g. minFrac = 0.05). Select data sets were run on XCMSOnline^64^, these included ST000062 and three of the eight data sets from ST000046. Additionally, only the negative ionization mode data for MTBLS352 (D1) was processable. For study D2, we used both the serum and the matching DBS samples together. Processing was parallelized using StarCluster (https://star.mit.edu/cluster/) on Amazon’s Elastic Compute Cloud (EC2).

Following feature extraction, output files were parsed along with additional author-provided metadata using the python jupyter notebook extracting_features.ipynb. Each study was analyzed independently; labels (0 for controls, 1 for cases) and metadata were extracted and mapped to the samples from the XCMS output. Select multiclass studies were reduced to binary problems (MTBLS315, MTBLS579, ST000381, ST000385, ST000421, ST000396 and ST000888). For ST000381, all categories other than healthy (i.e. modest, intermediate, and severe) were considered cases. For non-reducible multiclass data sets, we created data sets for each possible one-versus-one comparison. When replicates were included for a sample, only one was arbitrarily kept, minimizing bias imposed on the data.

To correct for batch effects, all data sets were transformed using the percentile normalization strategy by Gibbons et al.^51^. If batch information could be determined, each batch was normalized separately and then combined; lacking such information, normalization was applied to the full data set (batch_correction.ipynb). Prior to normalization, missing or < 1 peak intensities were set to 1, followed by binary log transformation of the data. For select author-data sets that were already log transformed, this normalization was not performed.

Within study data set combination was performed using combining_datasets_internal_to_study.ipynb. A manually curated list of combinable data sets was created and the feature matrices were concatenated, ensuring that samples for a single individual across data sets were combined into a single feature vector.

### Adduct and isotope determination

Using only the high resolution MS data sets (Table S1), we calculated the mass difference between each statistically significant feature and all others. A feature was considered an adduct or isotope if both features possessed retention times < 15 s apart, the mass difference was not zero, and was in a defined range. Significant features were assumed to be either [M−H]^−^ or [M + H]^+^ ions. All mass windows were differences relative to one of the two states:

[0.994, 1.012] = 1 ^13^C, [1.998, 2.016] = 2 ^13^C.

Positive ionization:

[21.975, 21.985] = Na^+^ , [37.954, 37.962] = K^+^

Negative ionization:

[35.969, 35.985] = Cl^−^, [18.006, 18.016] = –H_2_O–H.

### Machine learning

Model training was performed using either noncombined_model_training.ipynb or combining_datasets_internal_to_study.ipynb for the non-combined data sets and the combined data sets respectively. For each, L1-LR (sklearn.linear_model.LogisticRegressionCV, scoring = roc_auc, tol = 0.0001, intercept_scaling = 1 and max_iter = 500) and PLS-DA models (sklearn.cross_decomposition.PLSRegression, default settings) were trained using a fivefold outer (sklearn.model_selection.StratifiedKFold), threefold inner stratified and nested cross validation protocol. Inner cross validation selected either the L1-regularization parameter or the number of components used by the PLS-DA model (C = [2, 5, 20, 50, 100], sklearn.model_selection.GridSearchCV). RF, SVM, kNN and NB models were trained in the same manner with the following parameters: RF (sklearn.ensemble.RandomForestClassifier) with n_estimators = 500, SVM (sklearn.svm.SVC) with kernel = linear in a GridSearchCV with parameters gamma = [1e−3, 0.01, 0.1, 1] and C = [0.01, 0.1, 1, 10, 100], default GaussianNB (sklearn.naive_bayes.GaussianNB) parameters and n_neighbors = [1, 3, 5, 10] for a GridSearchCV wrapped kNN model (sklearn.neighbors.KNeighborsClassifier). Test performance was assessed on the independent fold in the outer cross-validation loop. To ensure that performance was not altered by data splits, this protocol was repeated independently 30 times on full data shuffles (sklearn.utils.shuffle) from which a data set average AUC (sklearn.metrics.roc_curve), standard deviation and 95% confidence interval was calculated (using the average number of test cases for sample size to reflect the larger uncertainty in the smaller data sets). The last model training was saved for analysis. Additional performance metrics including precision, accuracy, sensitivity, specificity and Mathews correlation coefficient were calculated using the following functions from the sklearn.metrics module: balanced_accuracy_score, matthews_corrcoef, confusion_matrix (used to obtain false positive, false negative, true positive and true negative values, used to calculate precision, sensitivity and specificity). These metrics were calculated for each fold and averaged over all individual model trainings for a given classifier.

### Statistical analysis and feature selection

P-values were corrected for multiple testing using the Benjamini–Hochberg False Discovery Rate (BH-FDR) method applied to the results of a MW-U test (statsmodels.stats.multitest.multipletests and scipy.stats.mannwhitneyu, giving Q-values) since all multiclass problems were reduced to multiple one-to-one comparisons. To determine the number of significant features for a given data set, Q-values were calculated using the full data matrix and all values < 0.05 were considered significant. However, to train models using only statistically significant features, but not have information from the test set leak into the model training, we calculated Q-values internal to the model training loop after the fivefold stratified data splitting; significant features found in the training step were subsequently used for testing and often differed across folds and data set shuffles. To train models using up to the *x* most significant features, a similar protocol was followed and only the *x* most significant features (largest negative log Q-values) were retained for model training and testing. If fewer than *x* features were found significant, only those features were used, despite not reaching the specific value of *x*. For models trained with only non-significant features (Q-values ≥ 0.05), all significant features from the complete full data set were removed prior to training. Features belonging to adducts and isotopes were similarly removed prior to training; such models were only built for the non-combined, high resolution MS studies. If any data set possessed 0 features, an AUC of 0.5 was recorded and training stopped. Overfit models with test AUC values less than 0.5 were retained. For each data set, feature coefficients from trained models were averaged.

MW-U tests were used for the significant testing of: GC and LC, positive and negative ionization, C18 and HILIC, plasma and serum, as well as all one-to-one ‘all features versus *x* significant features’ comparisons. Feature enrichment was calculated for each percentile-normalized feature as the mean value in the cases divided by the mean value in the controls.

## Supplementary information

Supplementary Data

Supplementary Information

## Data Availability

All original mass spectrometry data used is freely available and listed in Table S1. Packaged data set python pickle (.pkl) and input features with matched label files (.csv), along with model training output .csv and trained model .pkl files can be found on Zenodo (10.5281/zenodo.3885865). Model training results, performance metrics and general study metadata can be found in the accompanying file Data_S1.xlsx.

## Code availability

All code (.sh, .py, .R, and .ipynb) can be found on the following Github repository: https://github.com/ethanev/Metabolomics_ML.
