# Supplementary material for: Predicting human health from biofluid-based metabolomics using machine learning
Source: Sci Rep. 2020 Oct 19;10:17635. doi: 10.1038/s41598-020-74823-1 (PMC7572502; doi:10.1038/s41598-020-74823-1)
Supplement: Supplementary file 2 — Supplementary Information [file 41598_2020_74823_MOESM2_ESM.pdf]

Supplementary Materials for

Predicting human health from biofluid-based metabolomics using machine learning

Ethan D. Evans[1], Claire Duvallet[1,†], Nathaniel D. Chu[1], Michael K. Oberst[2], Michael A. Murphy[1,2], Isaac Rockafellow[1,‡], David Sontag[2], Eric J. Alm[1*]*

[1]Department of Biological Engineering, Massachusetts Institute of Technology, Cambridge, Massachusetts, 02139, United States.

[2]CSAIL, Massachusetts Institute of Technology, Cambridge, Massachusetts, 02139, United States.

† Current Affiliation: Biobot Analytics, Somerville, Massachusetts 02143, United States.
‡ Current Affiliation: Superpedestrian, Cambridge, Massachusetts 02139, United States.

Corresponding authors
*Eric J. Alm (ejalm@mit.edu, 617-253-2726)
*David Sontag (dsontag@mit.edu, 617-258-0625)

This file includes:

Supplementary tables and figures
References

Supplementary tables and figures

Table S1. Project identification or DOI for studies used. For data acquired from Metabolomics Workbench, this data is available at the NIH Common Fund's Metabolomics Data Repository and Coordinating Center (supported by NIH grant, U01-DK097430) website, the Metabolomics Workbench, http://www.metabolomicsworkbench.org. 'High res' refers to high resolution mass spectrometry used. ‡Refers to data sets without published accompanying manuscript.

Project or paper DOI	Project ID or Accession	High res	ID
https://doi.org/10.1093/gigascience/gix087	MTBLS408[1]	Yes	I1
10.21228/M8FH50	ST000763‡	Yes	I2
https://doi.org/10.1002/ijc.28491	MTBLS92[2]	Yes	B1
10.21228/M86K6W	ST000355, ST000356[3]	-	B3 / B2
10.21228/M8QM3R	ST000918[4]	Yes	B4
10.1021/pr500494u	ST000284[5]	-	B5
https://doi.org/10.1021/pr300673x	MTBLS19[6]	Yes	B7
http://dx.doi.org/10.1016%2Fj.aca.2012.07.013	MTBLS17[7]	Yes	B9
https://doi.org/10.1371/journal.pone.0127299	MTBLS105[8]	-	B8
0.21228/M8VH5Q	ST000865[9]	-	B6
10.21228/M8RC8J	ST000389, ST000388[10]	Yes	B11
https://doi.org/10.1158/0008-5472.CAN-14-0109	MTBLS28[11]	Yes	B14
10.21228/M8T60D	ST000392[12]	-	B12
10.21228/M80W30	ST000368, ST000369, ST000385, ST000386[13]	-	B10
10.21228/M86G6V	ST000396[14] paper associated with general cohort	-	B13
10.1038/srep22525	Feng et al.[15]	Yes	C1
10.21228/M8NS3V	ST000329‡	Yes	G1
10.1186/s13073-016-0318-8	MTBLS279, MTBLS280, MTBLS253[16]	-	E1 / E2
10.21228/M8X96N	ST000888[17]	Yes	E3
10.21228/M8DP41	ST000578[18]	Yes	E4
http://dx.doi.org/10.1371%2Fjournal.pntd.0004480	MTBLS315[19]	Yes	E5
https://doi.org/10.1016/j.diagmicrobio.2016.03.012	MTBLS354[20]	Yes	E6
10.1371/journal.pntd.0006215	MTBLS579[21]	-	E7
https://doi.org/10.1093/gigascience/gix036	MTBLS352[22]	Yes	D1
10.21228/M88C86	ST000383[23]	-	D4
10.21228/M8J60X	ST000608[24]	Yes	D2
10.21228/M8D59R	ST000045[25]	Yes	D3
10.21228/M88G6G	ST000046[26]	Yes	A1
https://doi.org/10.1038/nm.3466	MTBLS72[27]	Yes	A2
10.21228/M8V88F	ST000062, ST000063‡	-	A3
10.1073/pnas.1603023113	MTBLS266[28]	Yes	F1
10.21228/M82K58	ST000450[29]	-	F2
10.21228/M8D60P	ST000381[30]	-	F3
10.4155/bio-2016-0108	MTBLS364[31]	Yes	F4
http://dx.doi.org/10.3390%2Fijms17091583	MTBLS358[32]	-	H1

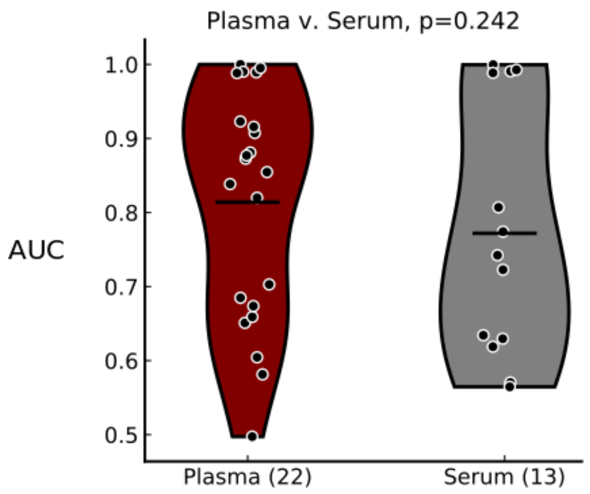

Figure S1. Plasma and serum-based studies display no statistically significant difference in AUC values. Values used are only from reprocessed studies with points representing individual data sets each randomly dispersed in the x-dimension for ease of viewing. Values in parentheses indicate the number of data sets in each category. P-value shown is from a MW-U test.

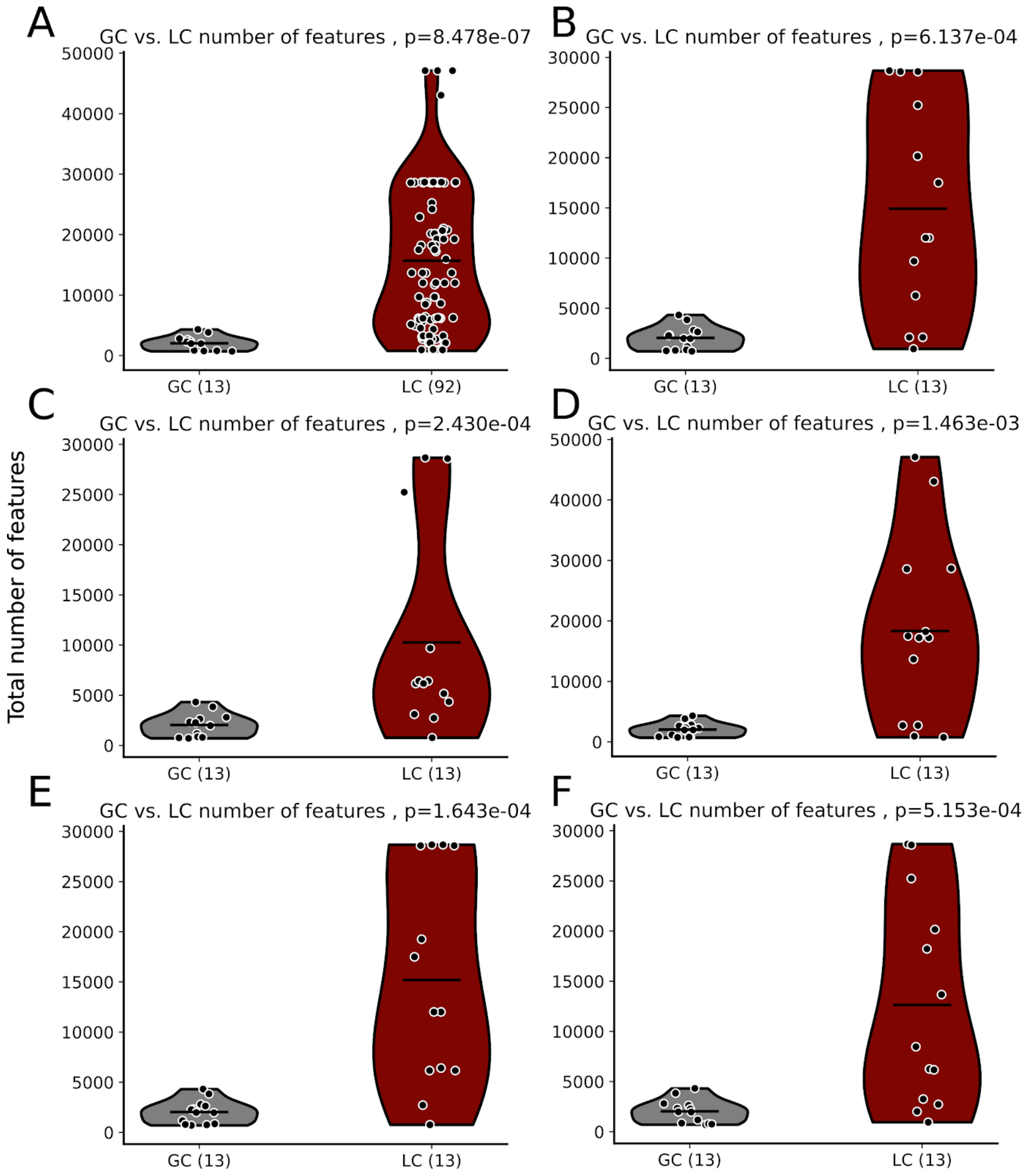

Figure S2. Reprocessed LC studies tend to possess more features than GC-based studies. (A) Comparison of all LC versus all GC studies in terms of the total number of features following XCMS processing. (B-F) Random sampling (with replacement) of the LC samples for balanced comparison. All P-values shown are MW-U tests. Values used are only from reprocessed studies with points representing individual data sets each randomly dispersed in the x-dimension for ease of viewing. Values in parentheses indicate the number of data sets in each category.

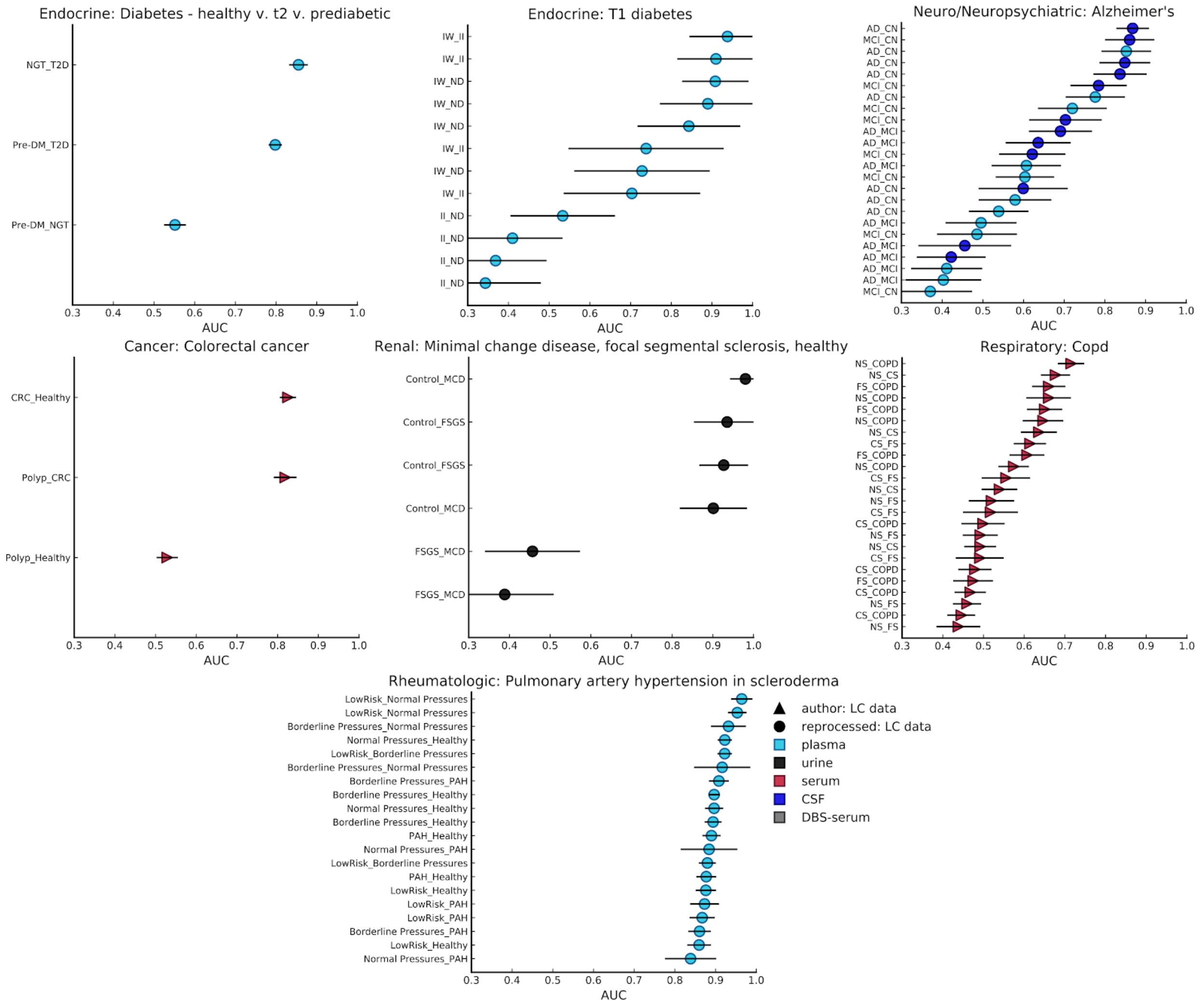

Figure S3. Low model AUC values are frequently due to the challenge of differentiating highly similar (or the same) health states. Shown are the results for individual data sets for all multiclass studies. These include two endocrine, and one of each of the following: Neuro/Neuropsychiatric, cancer, renal, respiratory and rheumatologic health states. When multiple of the same labels are provided this is due to distinct data sets internal to each study looking at the same classes but with different methods, instruments or other experimental parameters. Select abbreviations are as follow, NGT: healthy control, T2D: type II diabetes, Pre-DM: Pre-diabetes; IW: 8 hour insulin withdraw, II: Insulin injection following 8 hour withdraw, ND: no diabetes; AD: Alzheimer's, CN: cognitively normal, MCI: mild cognitive impairment; CRC: colorectal cancer; MCD: minimal change disease, FSGS: focal segmental glomerulosclerosis; NS: never smoker, CS: current smoker, COPD: Chronic Obstructive Pulmonary Disease, FS: former smoker; PAH: pulmonary artery hypertension.

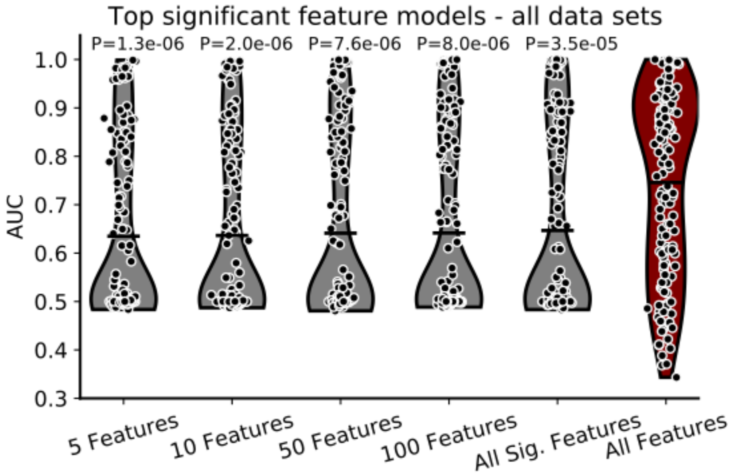

Figure S4. Models trained with all features outperform models using only significant features. All data sets were included in this plot for the significant feature (gray) subsets, this includes those data sets for which there are no significant features in the full data set but may have significant features following the five-fold splitting for which significant features are calculated on four out of the five folds and used for model training and testing (see main text methods). Data sets for which there were no features left after removing the non-significant features were assigned an AUC of 0.5, this explains the sudden stop in decreasing AUC observed for the significant feature models. The 'all feature' models were fully trained always and thus sometimes overfit on the training data leading to very low AUC values < 0.5. P-values correspond to MW-U tests for the model AUC values between each number of feature subsetting relative to the all feature data. Data points representing individual data sets are each randomly dispersed in the x-dimension for ease of viewing internal to each type of model built.

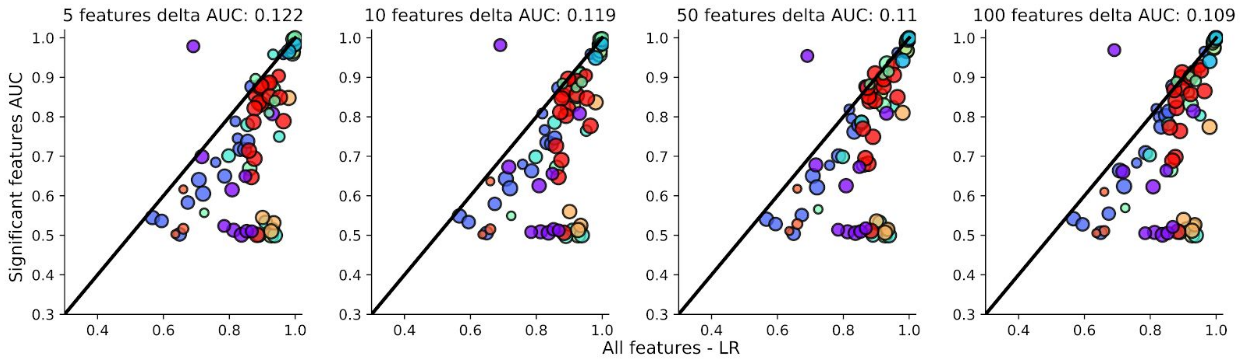

Figure S5. The AUC values for distinct data sets minimally changes when the 5–100 top significant features are used for model training. Plots show the AUC value using all features (X-axis) versus the AUC value of models using a select number of top features. Delta AUC values calculated by taking the mean of the differences between all features and the top feature models. Dot sizes are proportional to the log of the total number of features in the 'all features' data set for each plot. Colors correspond to health state categories from the main text.

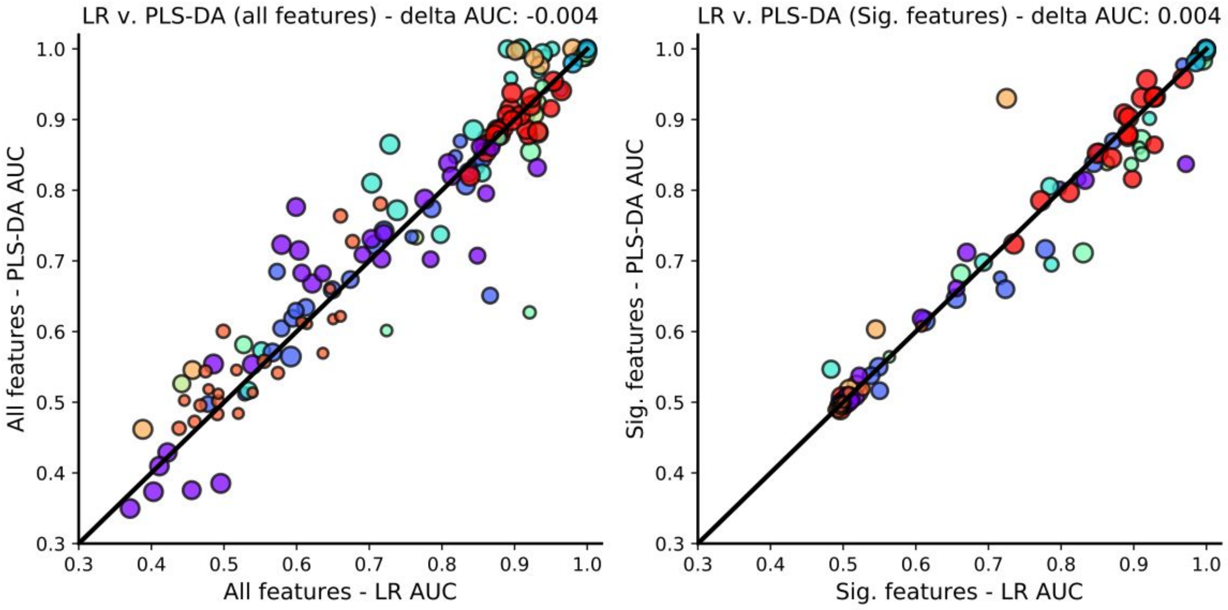

Figure S6. PLS-DA models perform on par with L1-LR models when trained on all features using AUC as a comparison metric. AUC scatter plot of models trained on individual data sets with the mean difference in AUC shown in the plot label (average of L1-LR AUC values minus the average of PLS-DA AUC values). Models trained using all features (left), models trained for data sets with only significant features (right). Dot sizes are proportional to the log of the total number of features for each plot. Colors correspond to health state categories from the main text.

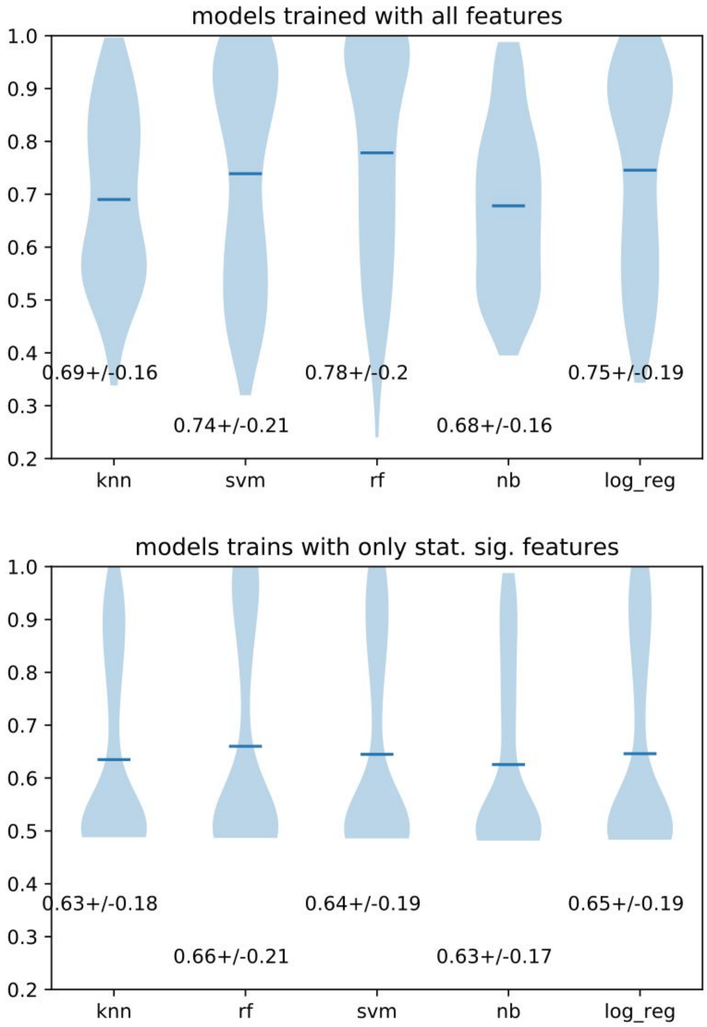

Figure S7. Comparison of machine learning models for both complete data sets and data sets with only statistically significant features. Knn = k-nearest neighbors, svm = support vector machine, rf = random forest, nb = naive bayes, plsda = partial least squares discriminant analysis, log_reg = L1-regularized logistic regression. Values show represent the mean and standard deviation of all of the data sets for a given model class, the mean is also plotted with a thick blue bar. The y-axis is AUC.

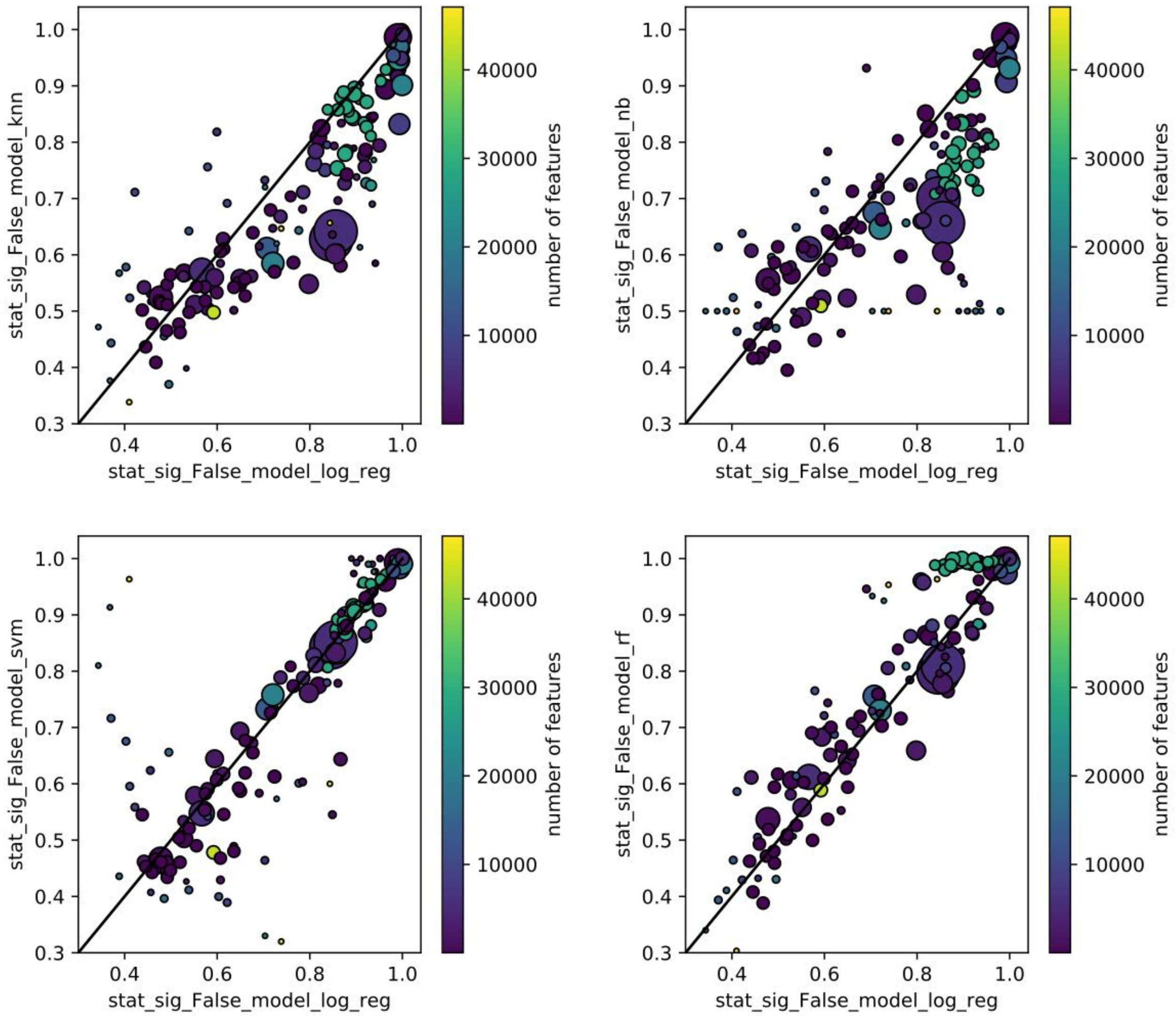

Figure S8. Comparison of logistic regression models and other machine learning models for each data set. AUC is plotted on both the X and Y axis for the labeled models. Models were trained with all features (ie 'stat_sig_False'). knn = k-nearest neighbors, svm = support vector machine, rf = random forest, nb = naive bayes, plsda = partial least squares discriminant analysis, log_reg = L1-regularized logistic regression. For each plot the circle size is proportional to the number of samples in the data set, color shows the number of features as indicated.

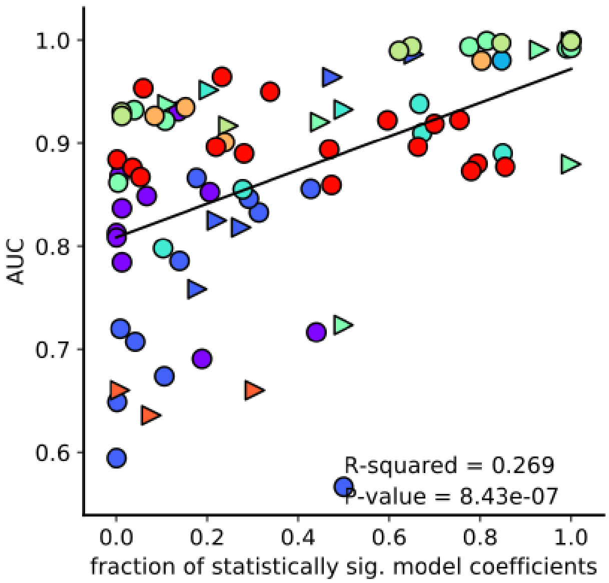

Figure S9. Correlation of AUC with fraction of statistically significant model coefficients for models built on data sets with at least one significant feature. Linear regression of the AUC value as a function of the fraction of statistically significant model coefficients. Regression performed with stats.linregress, giving R-squared and P-value. Colors correspond to health state categories from the main text.

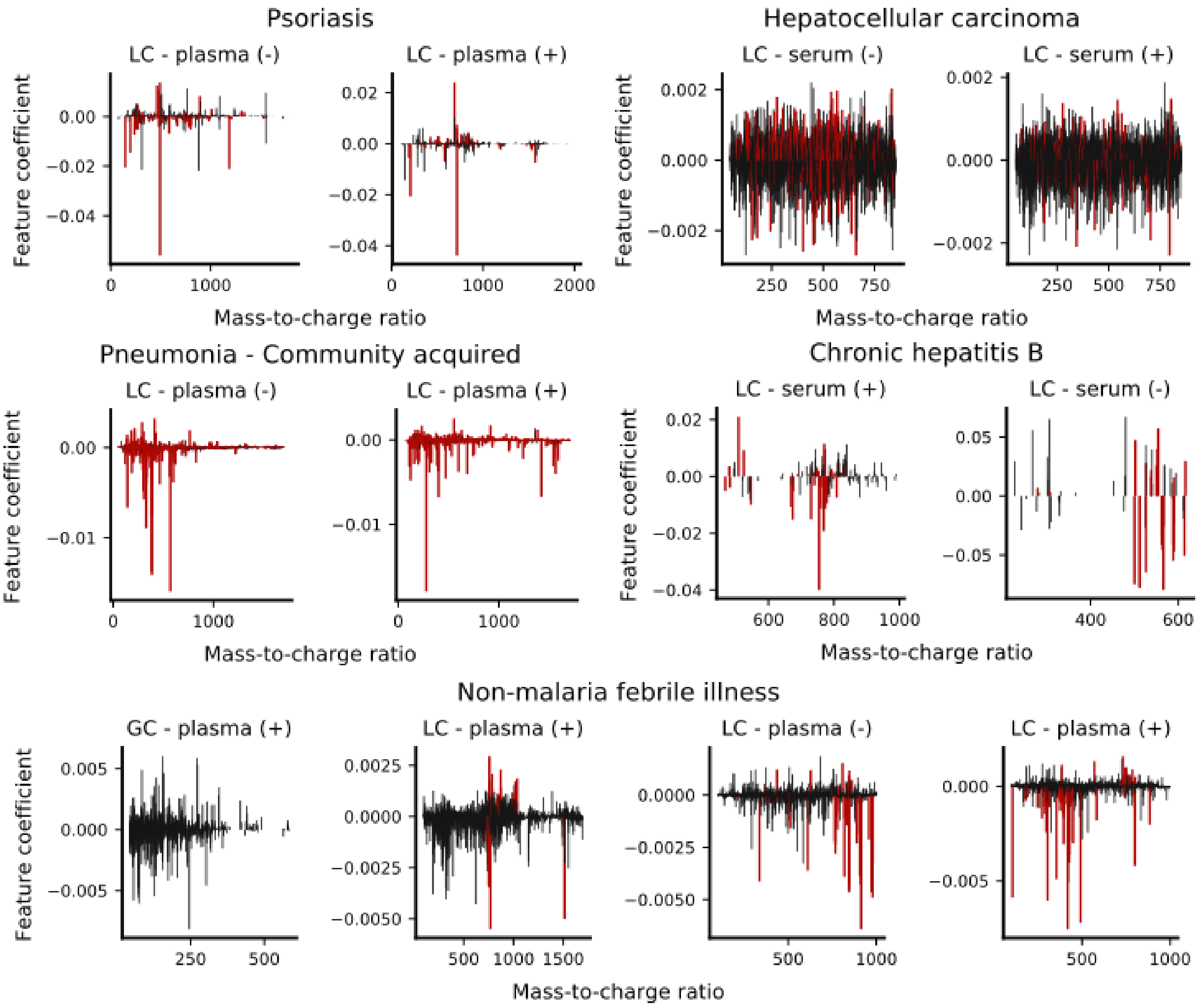

Figure S10. Mass-to-charge ratio versus model feature coefficient for individual data sets. Shown in red are the statistically significant features while black are not significant. Data sets (clockwise from upper left): Psoriasis - MTBLS408, Hepatocellular carcinoma - MTBLS17, Chronic hepatitis B - MTBLS279, Non-malaria febrile illness - MTBLS315, Pneumonia - MTBLS354. Note for the following data sets it was not possible to match mz values to the author supplied feature names: MTBLS358, MTBLS579, ST000284, ST000355, ST000356 and ST000450.

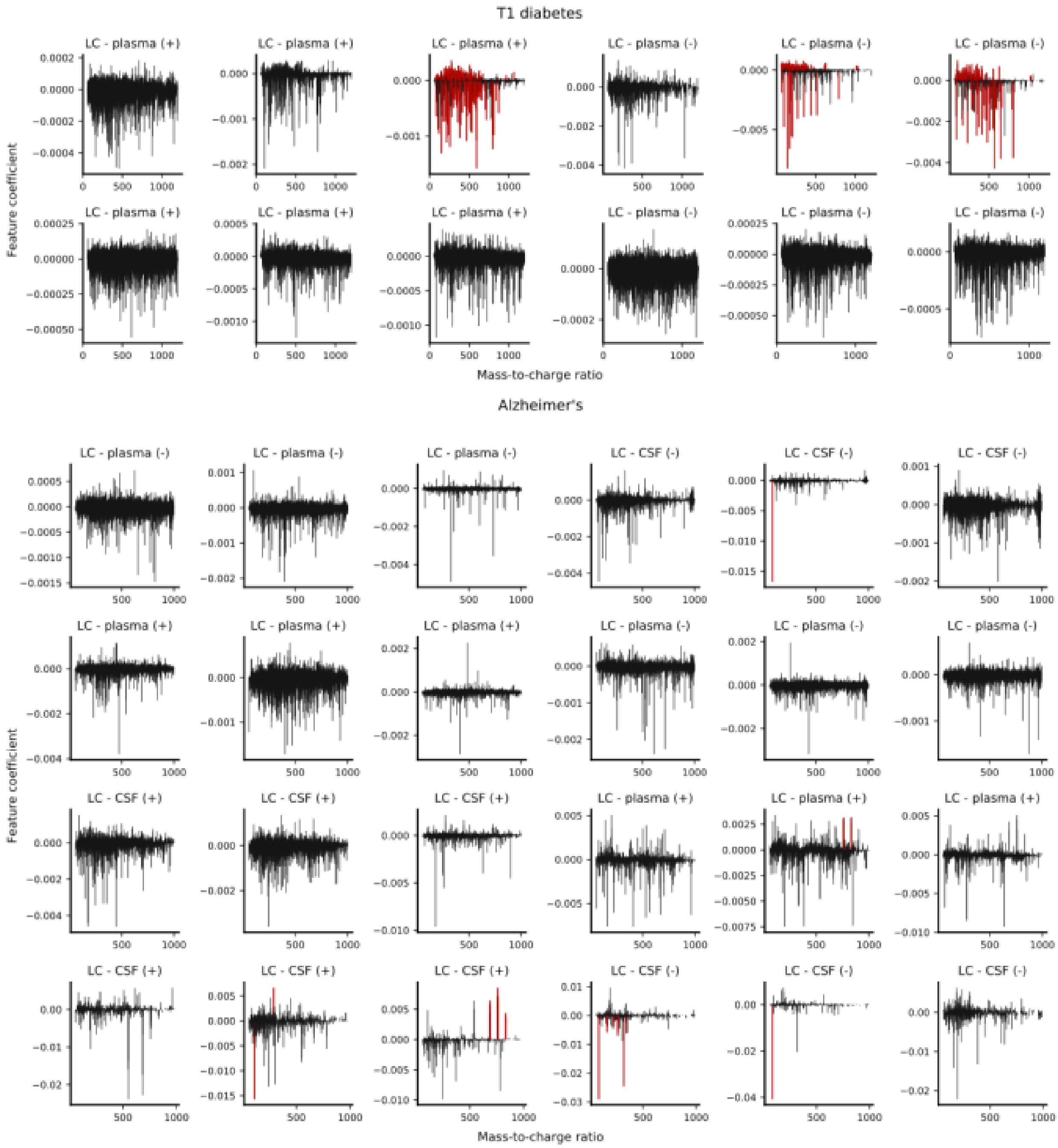

Figure S10 (continued). Mass-to-charge ratio versus model feature coefficient for individual data sets. Shown in red are the statistically significant features while black are not significant. Data sets (top to bottom): T1 diabetes - ST000045, Alzheimer's - ST000046.

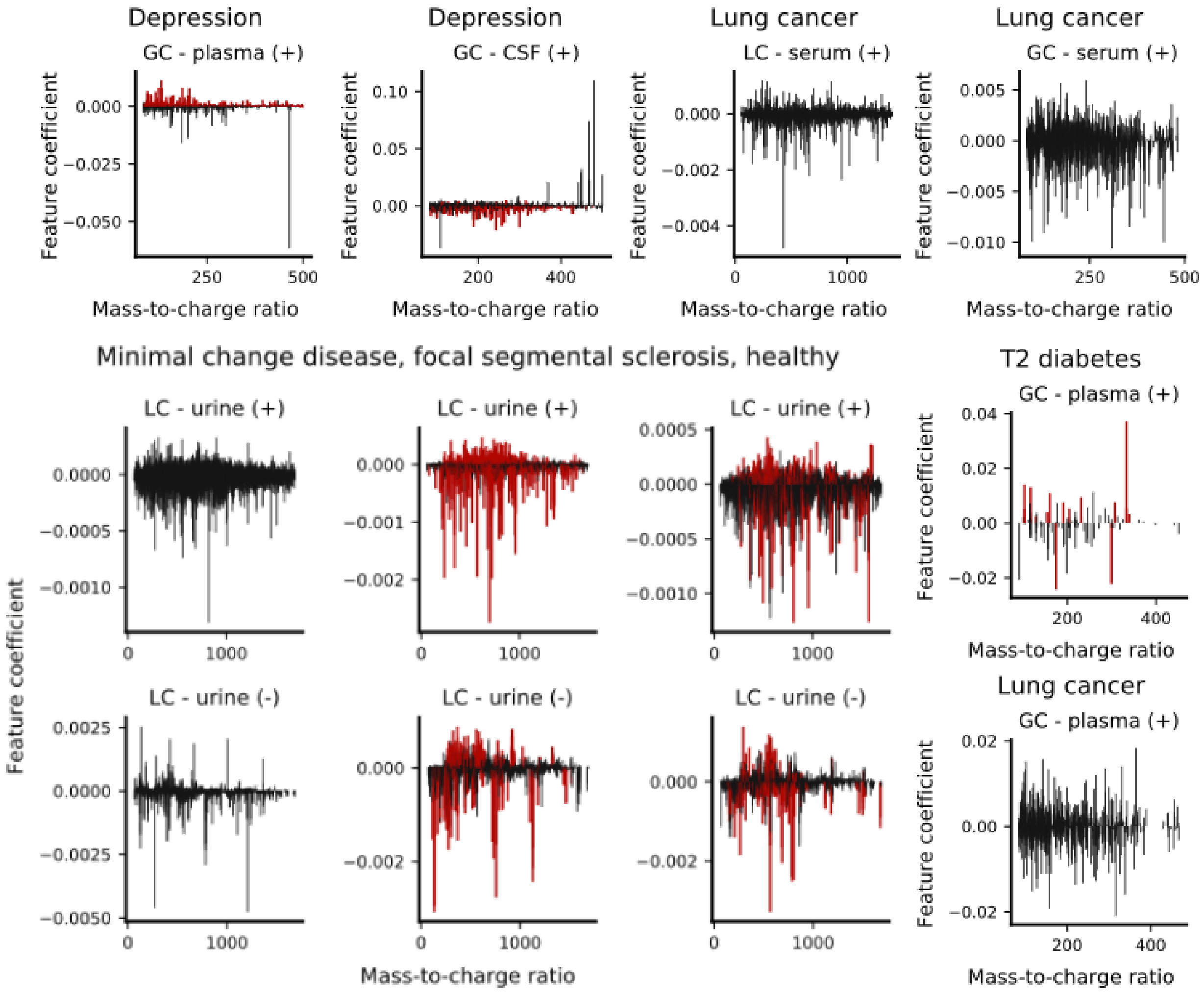

Figure S10 (continued). Mass-to-charge ratio versus model feature coefficient for individual data sets. Shown in red are the statistically significant features while black are not significant. Data sets (clockwise from upper left): Depression - ST00062, Depression - ST000063, Lung Cancer - ST000388, Lung Cancer - ST000389, T2 diabetes - ST000383, Lung cancer - ST000396, Minimal change disease - ST000329. ST000062 and 63 were considered part of the same study as were ST000388 and 389.

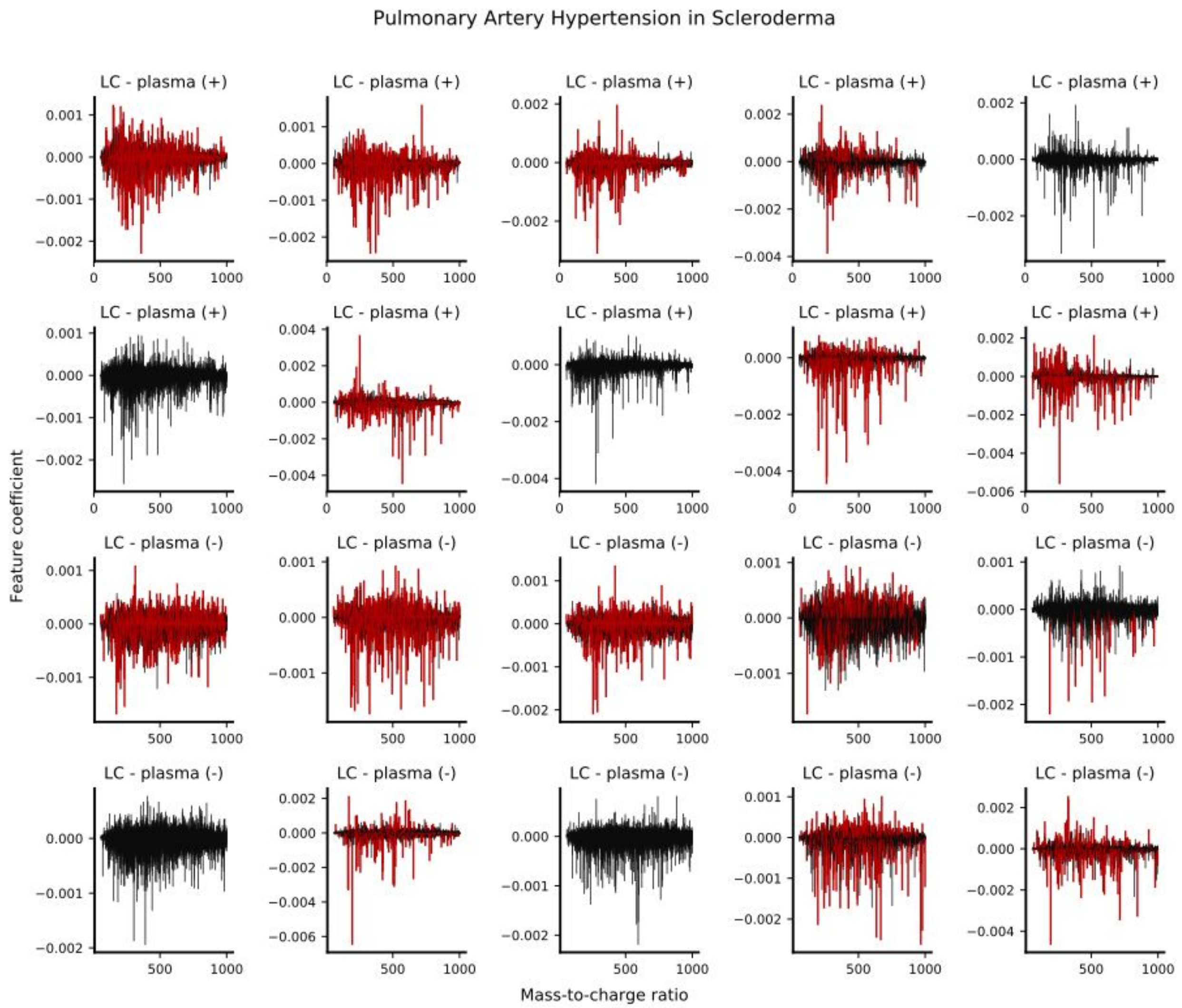

Figure S10 (continued). Mass-to-charge ratio versus model feature coefficient for individual data sets. Shown in red are the statistically significant features while black are not significant. Data set: ST000763.

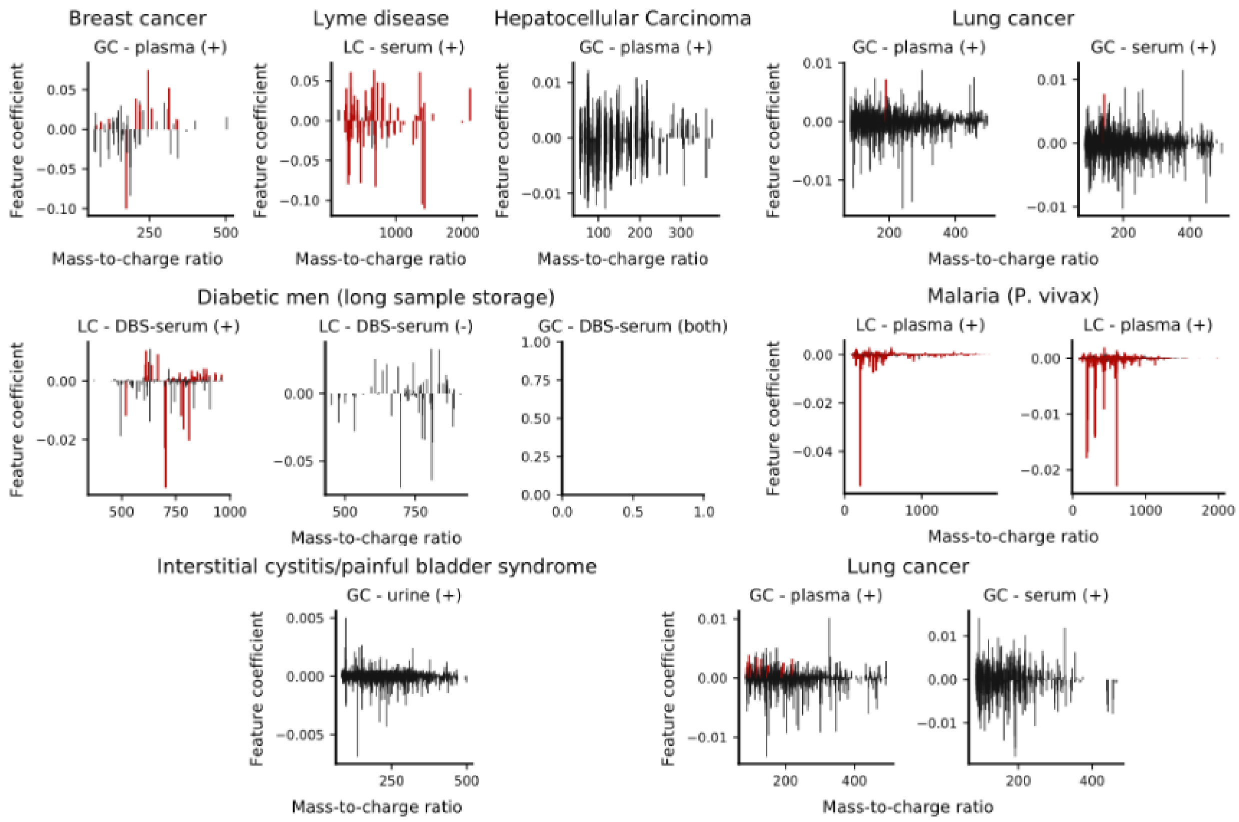

Figure S10 (continued). Mass-to-charge ratio versus model feature coefficient for individual data sets. Shown in red are the statistically significant features while black are not significant. Data sets (clockwise from upper left): Breast cancer - ST000918, Lyme disease - ST0008888, Hepatocellular carcinoma - ST000865, Lung cancer - ST000385, Malaria - ST000578, Lung cancer - ST000392, Interstitial cystitis - ST000381, Diabetic men - ST000608. Empty GC plot for the Diabetic men study is due to the inability to match features to mz values due to authors reporting only compound names and not mz values observed, while the LC data had matched rt and mz for each feature.

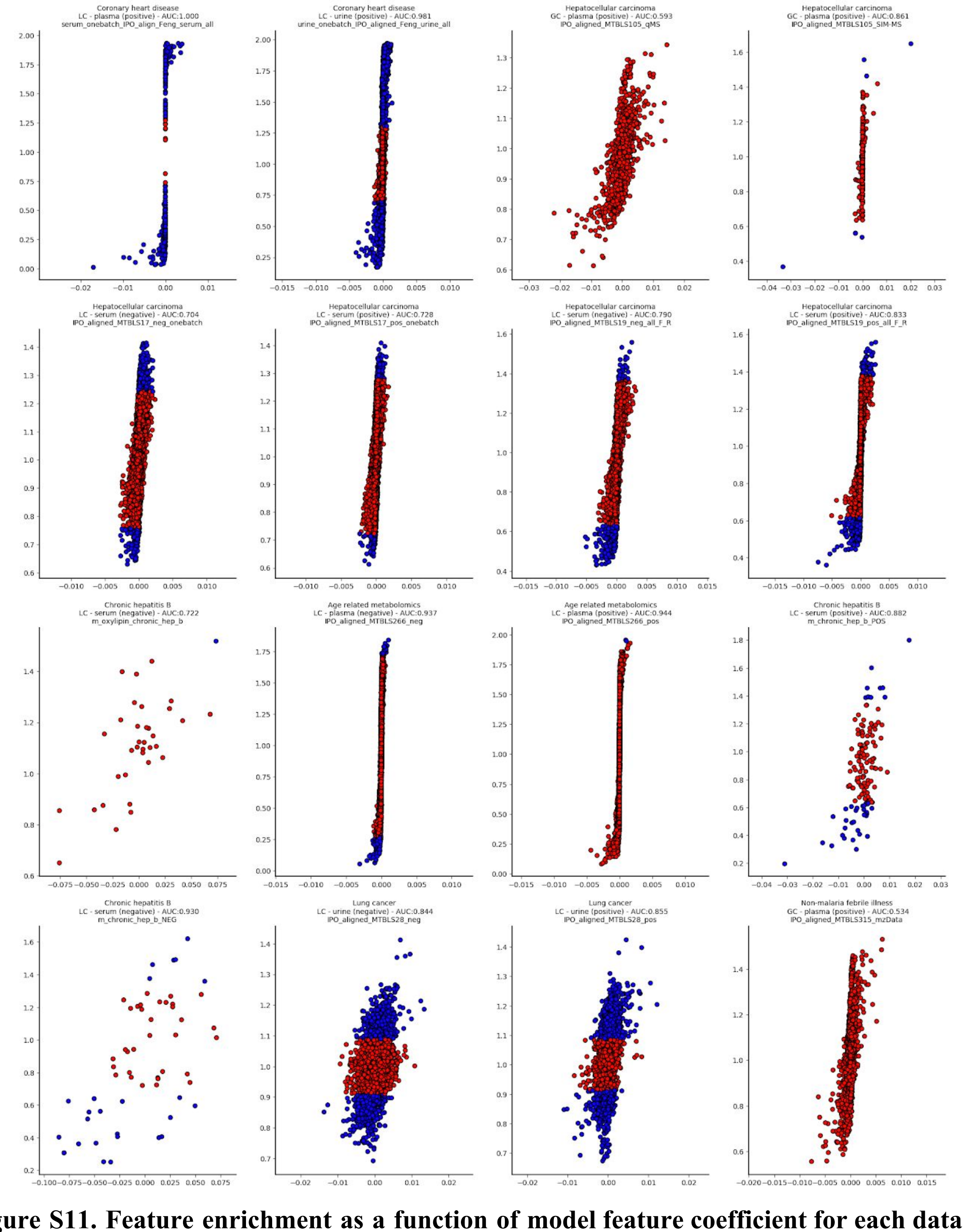

Figure S11. Feature enrichment as a function of model feature coefficient for each data set for all studies. Enrichment calculated as the average feature intensity in case divided by the average feature intensity in control. Red dots represent non-significant features while blue dots are statistically significant (P-value < 0.05, FDR-corrected MW-U test). The x-axis depicts the average model feature coefficient and the y-axis if the fold enrichment in case relative to control.

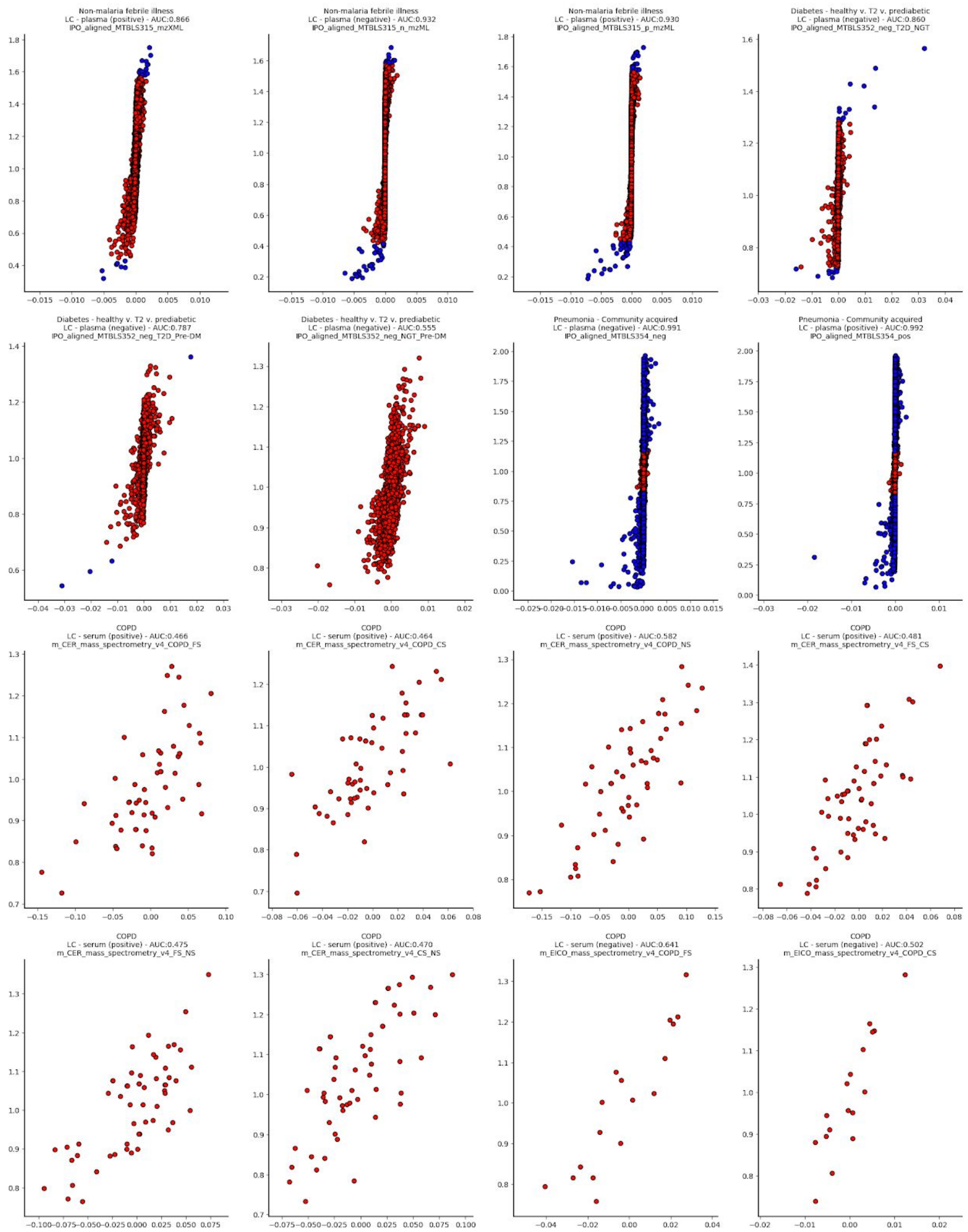

Figure S11 (continued). Feature enrichment as a function of model feature coefficient for each data set for all studies. Enrichment calculated as the average feature intensity in case divided by the average feature intensity in control. Red dots represent non-significant features while blue dots are statistically significant (P-value < 0.05, FDR-corrected MW-U test). The x-axis depicts the average model feature coefficient and the y-axis if the fold enrichment in case relative to control.

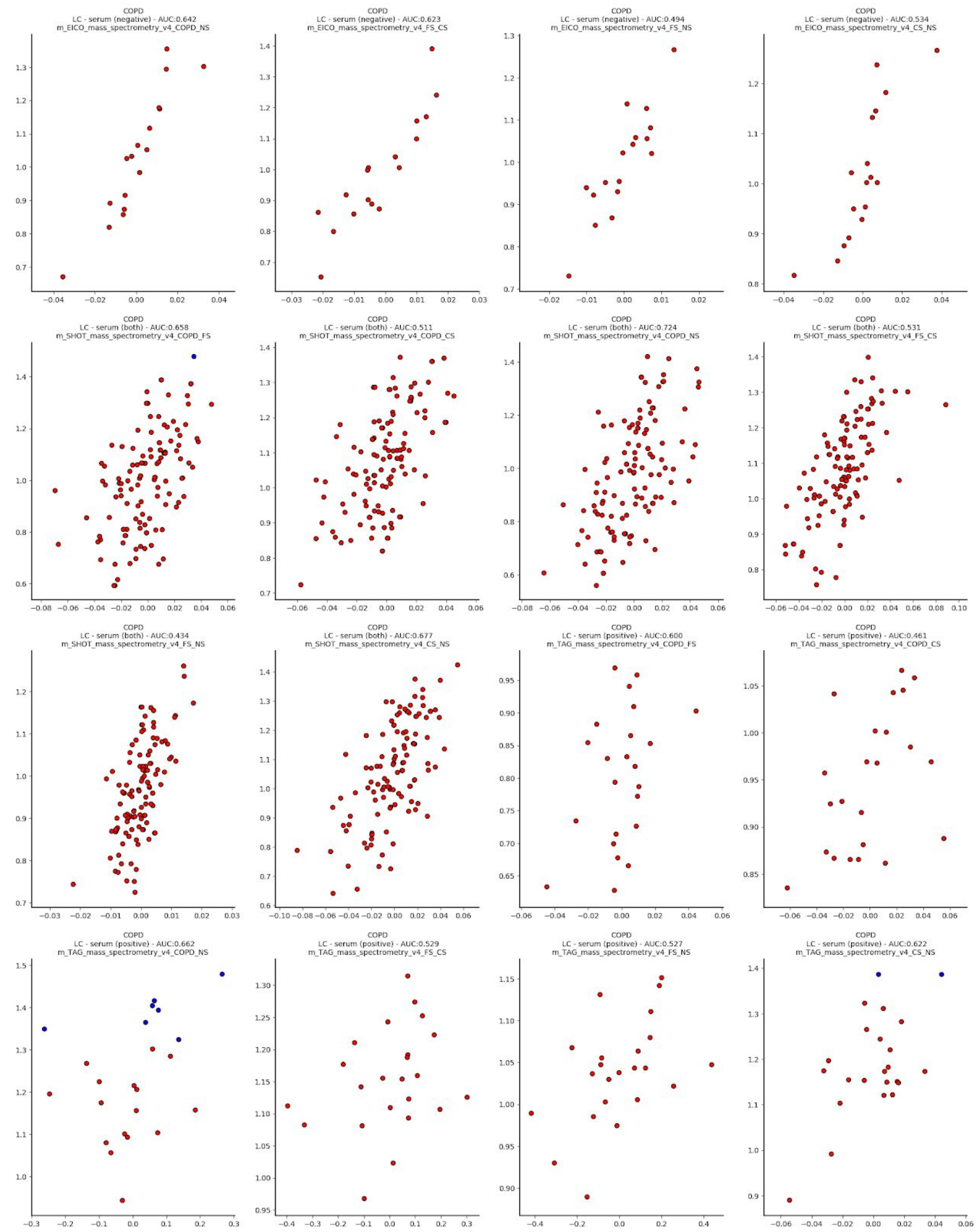

Figure S11 (continued). Feature enrichment as a function of model feature coefficient for each data set for all studies. Enrichment calculated as the average feature intensity in case divided by the average feature intensity in control. Red dots represent non-significant features while blue dots are statistically significant (P-value < 0.05, FDR-corrected MW-U test). The x-axis depicts the average model feature coefficient and the y-axis if the fold enrichment in case relative to control.

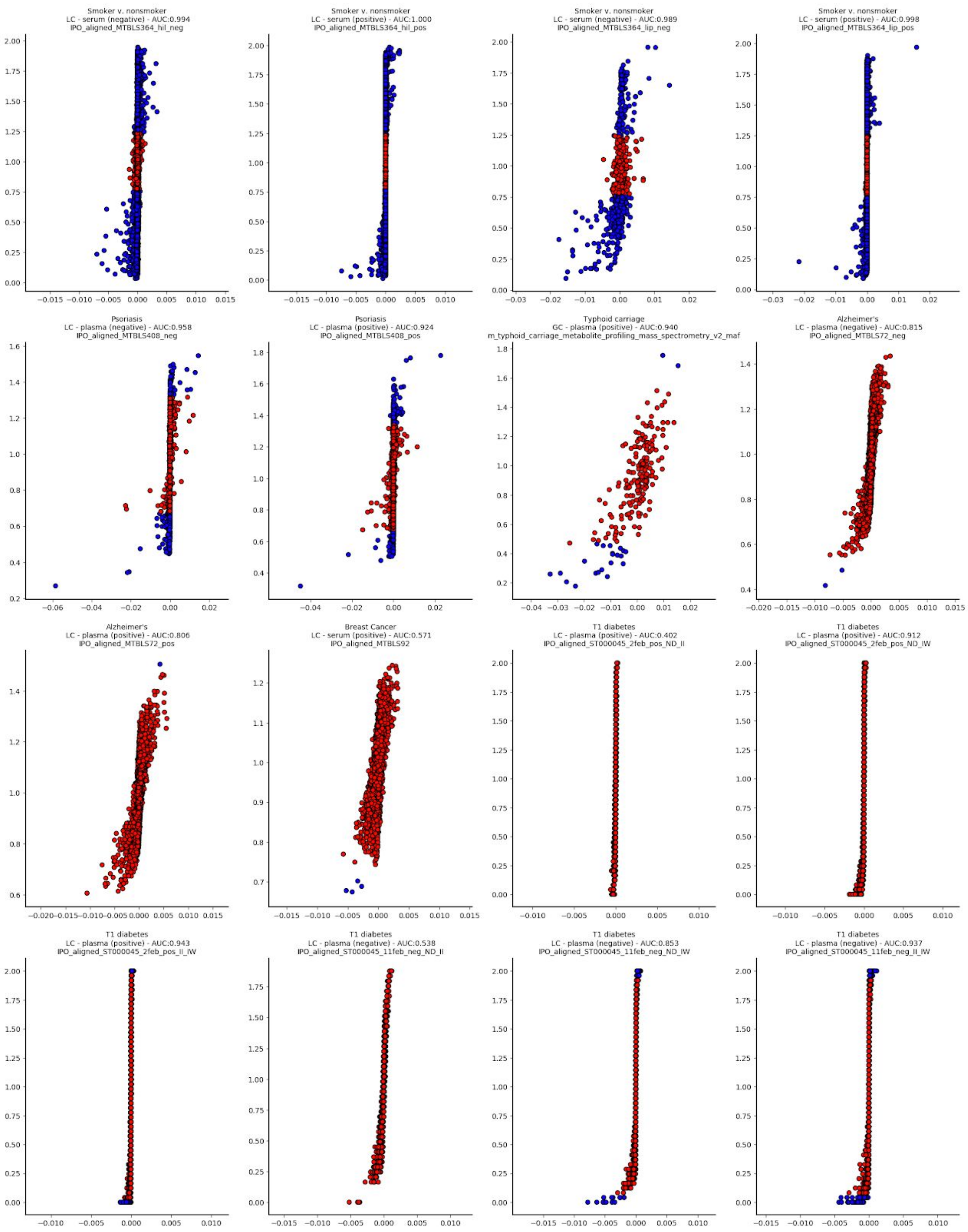

Figure S11 (continued). Feature enrichment as a function of model feature coefficient for each data set for all studies. Enrichment calculated as the average feature intensity in case divided by the average feature intensity in control. Red dots represent non-significant features while blue dots are statistically significant (P-value < 0.05, FDR-corrected MW-U test). The x-axis depicts the average model feature coefficient and the y-axis if the fold enrichment in case relative to control.

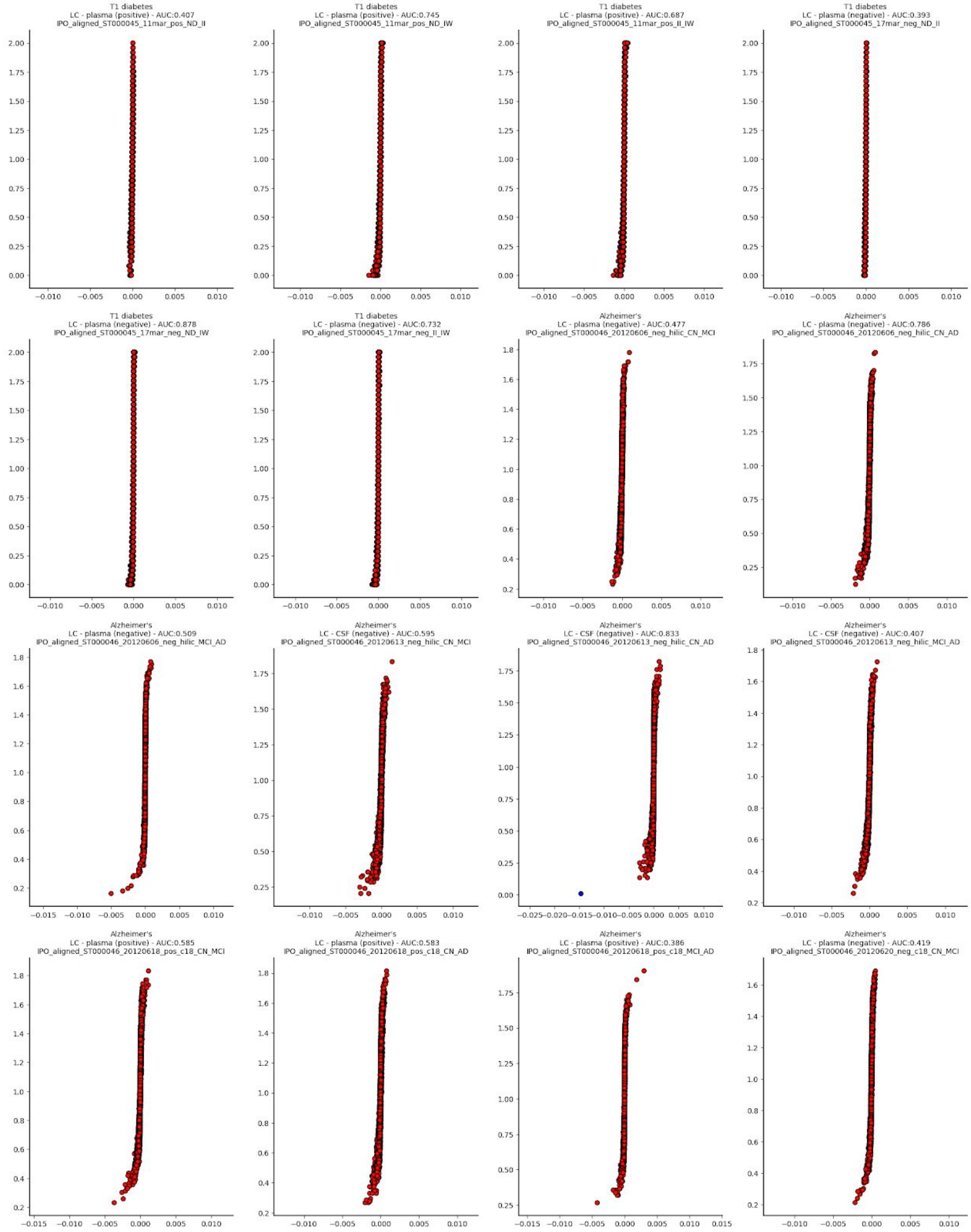

Figure S11 (continued). Feature enrichment as a function of model feature coefficient each data set for all studies. Enrichment calculated as the average feature intensity in case divided by the average feature intensity in control. Red dots represent non-significant features while blue dots are statistically significant (P-value < 0.05, FDR-corrected MW-U test). The x-axis depicts the average model feature coefficient and the y-axis if the fold enrichment in case relative to control.

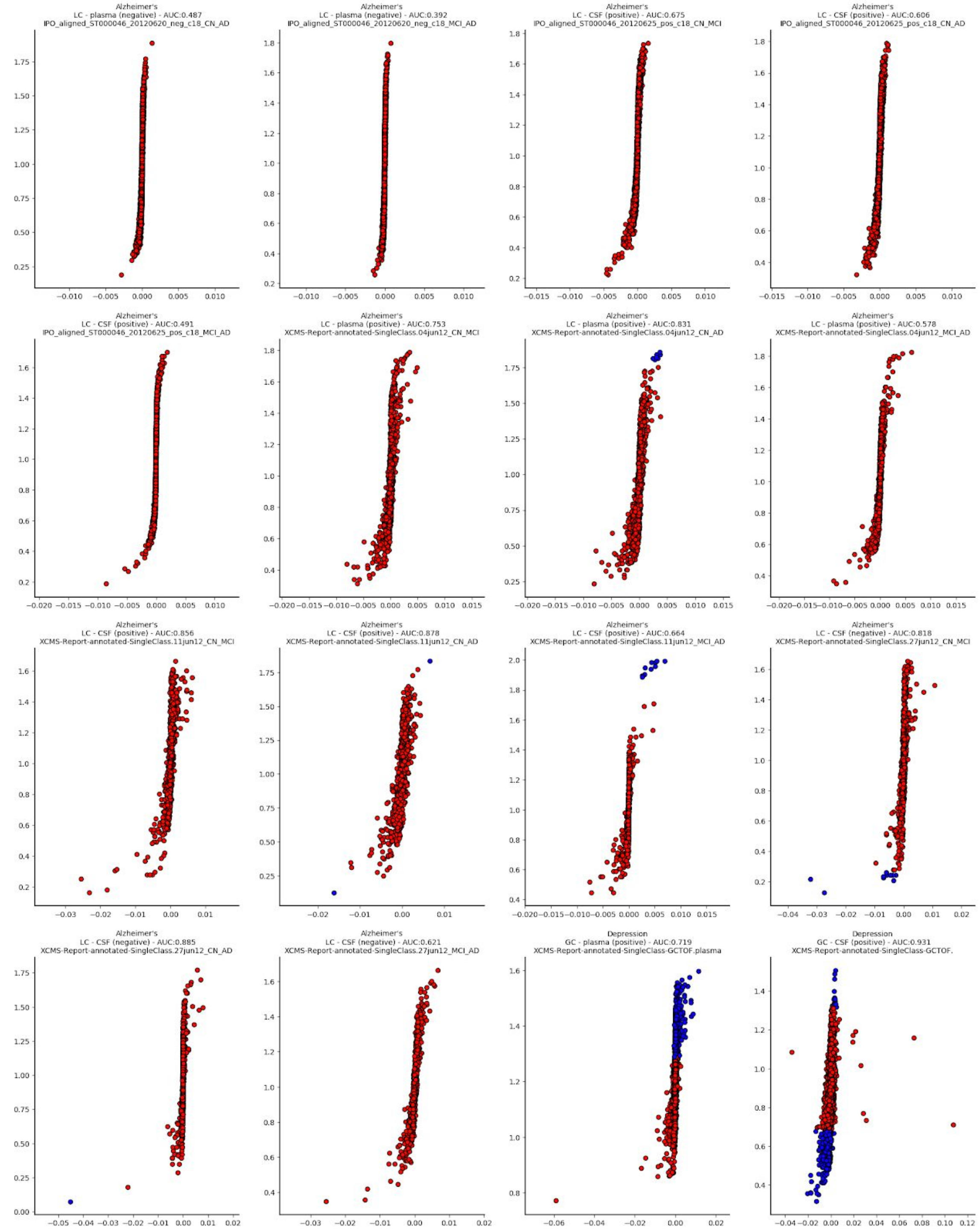

Figure S11 (continued). Feature enrichment as a function of model feature coefficient each data set for all studies. Enrichment calculated as the average feature intensity in case divided by the average feature intensity in control. Red dots represent non-significant features while blue dots are statistically significant (P-value < 0.05, FDR-corrected MW-U test). The x-axis depicts the average model feature coefficient and the y-axis if the fold enrichment in case relative to control.

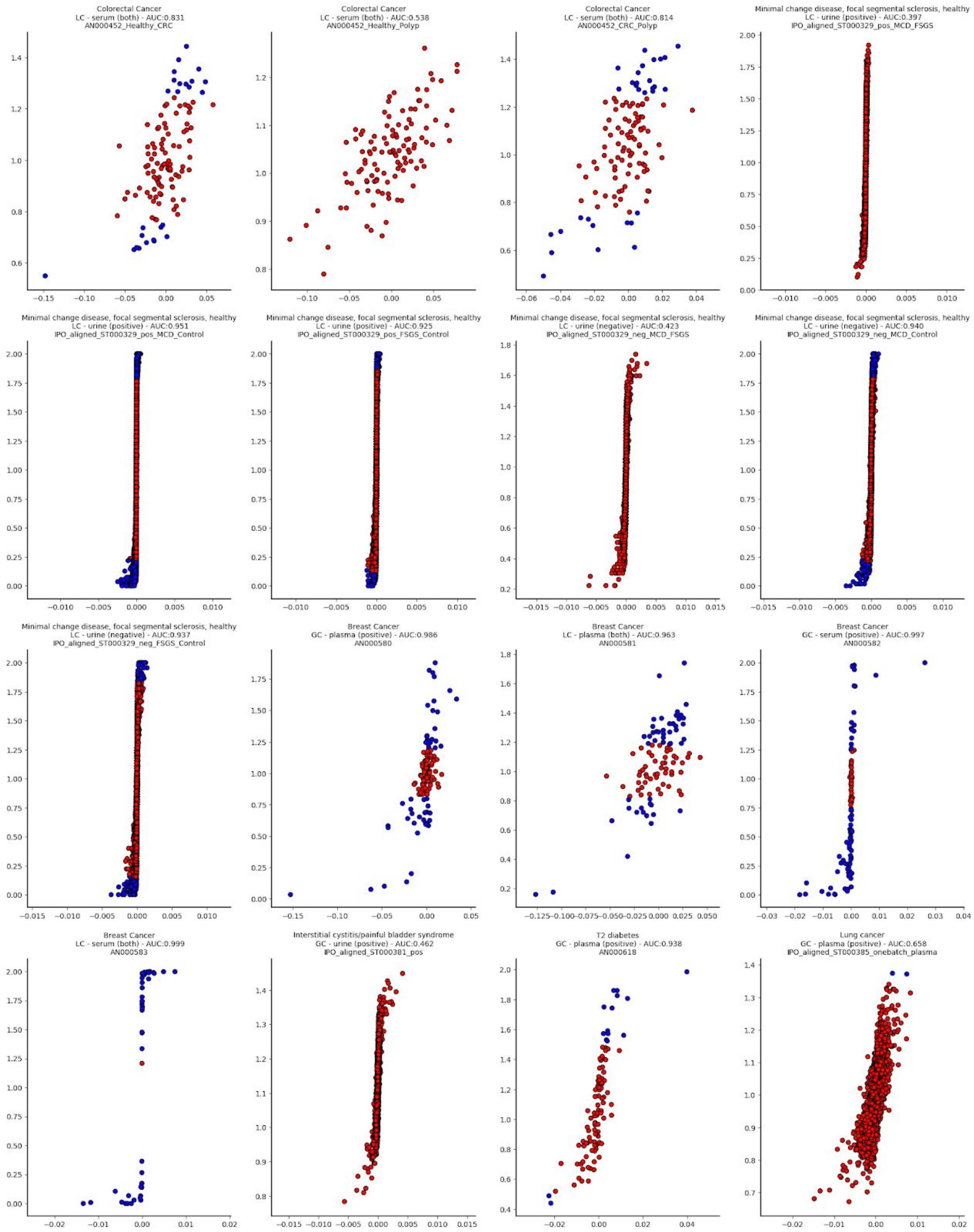

Figure S11 (continued). Feature enrichment as a function of model feature coefficient each data set for all studies. Enrichment calculated as the average feature intensity in case divided by the average feature intensity in control. Red dots represent non-significant features while blue dots are statistically significant (P-value < 0.05, FDR-corrected MW-U test). The x-axis depicts the average model feature coefficient and the y-axis if the fold enrichment in case relative to control.

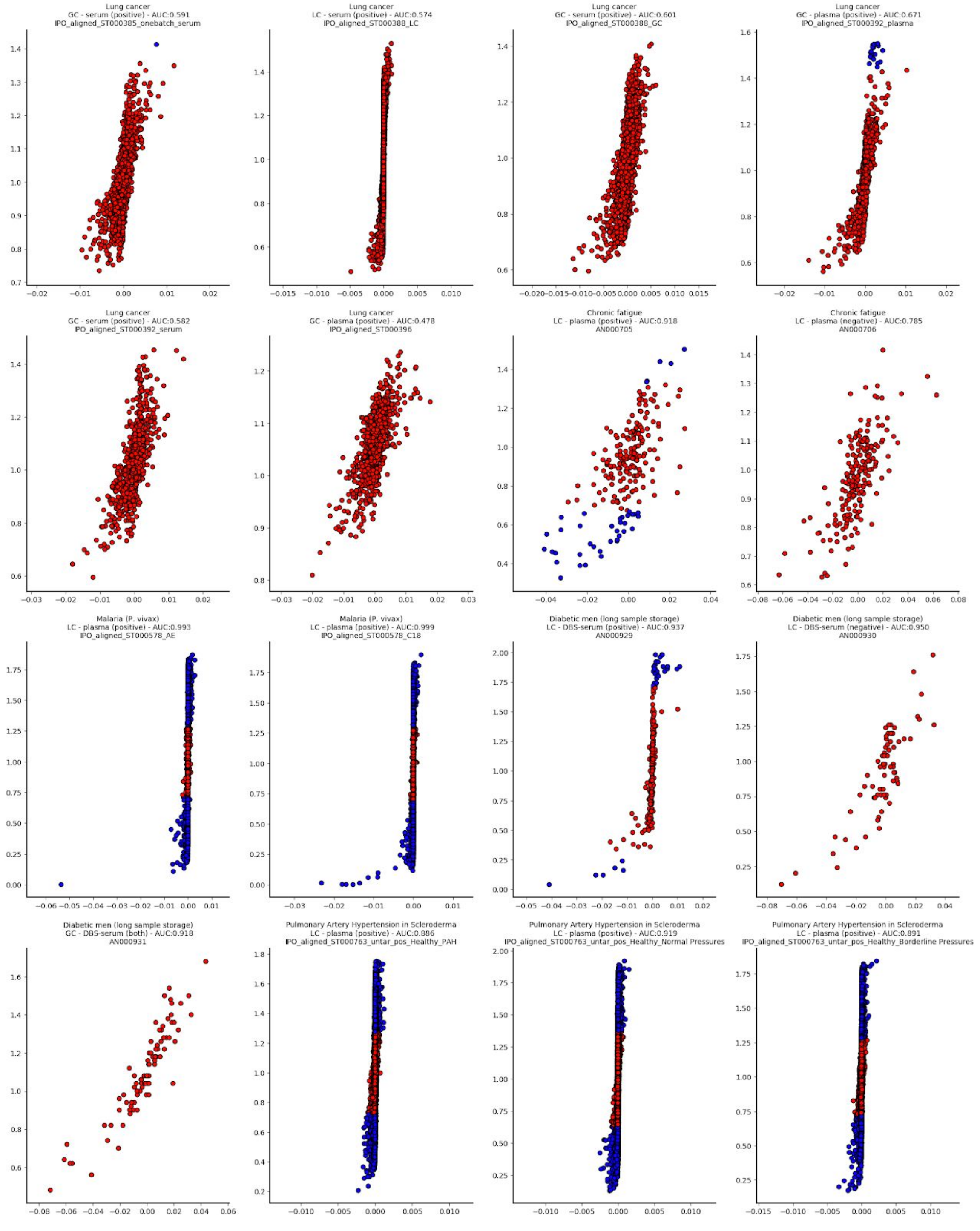

Figure S11 (continued). Feature enrichment as a function of model feature coefficient each data set for all studies. Enrichment calculated as the average feature intensity in case divided by the average feature intensity in control. Red dots represent non-significant features while blue dots are statistically significant (P-value < 0.05, FDR-corrected MW-U test). The x-axis depicts the average model feature coefficient and the y-axis if the fold enrichment in case relative to control.

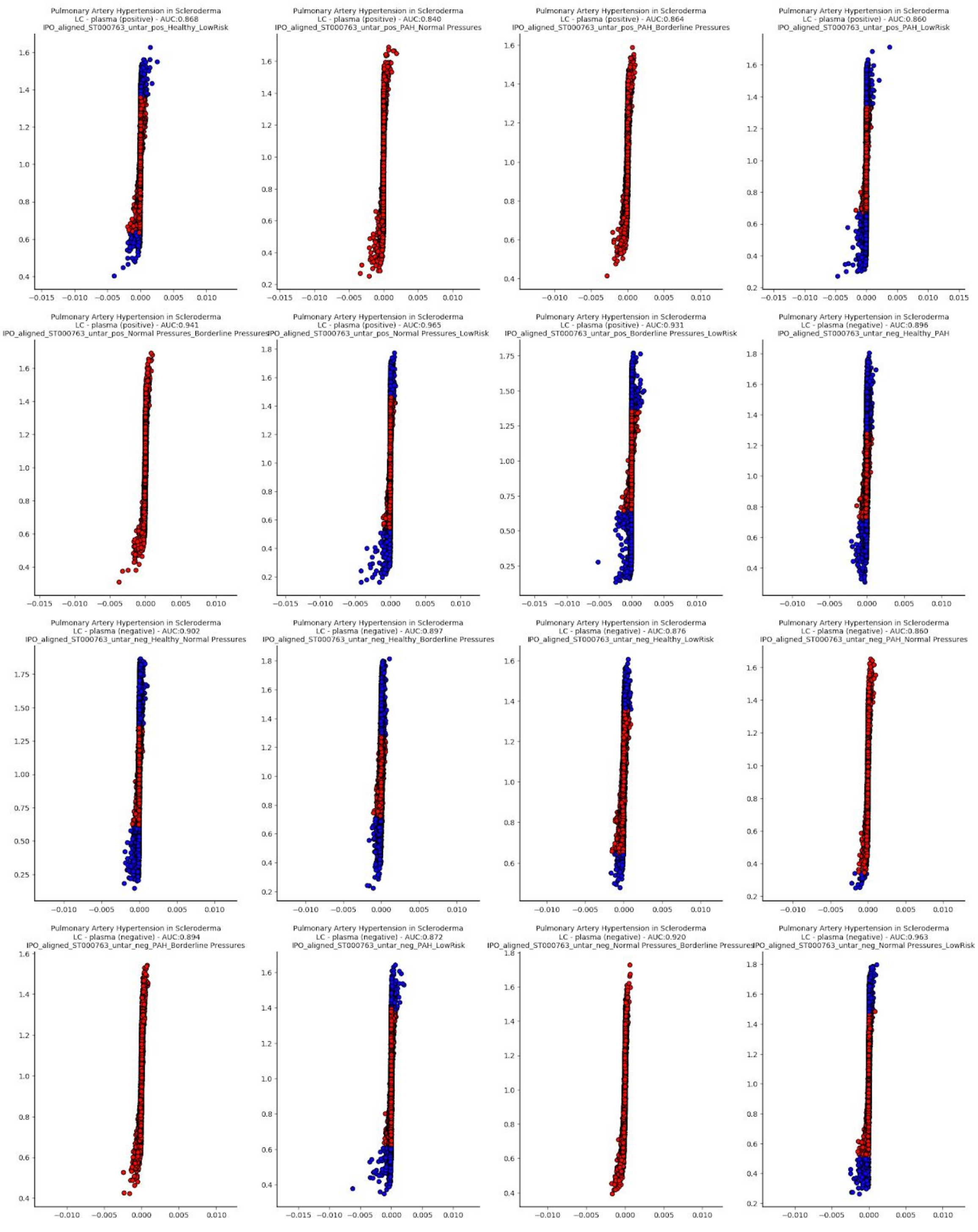

Figure S11 (continued). Feature enrichment as a function of model feature coefficient each data set for all studies. Enrichment calculated as the average feature intensity in case divided by the average feature intensity in control. Red dots represent non-significant features while blue dots are statistically significant (P-value < 0.05, FDR-corrected MW-U test). The x-axis depicts the average model feature coefficient and the y-axis if the fold enrichment in case relative to control.

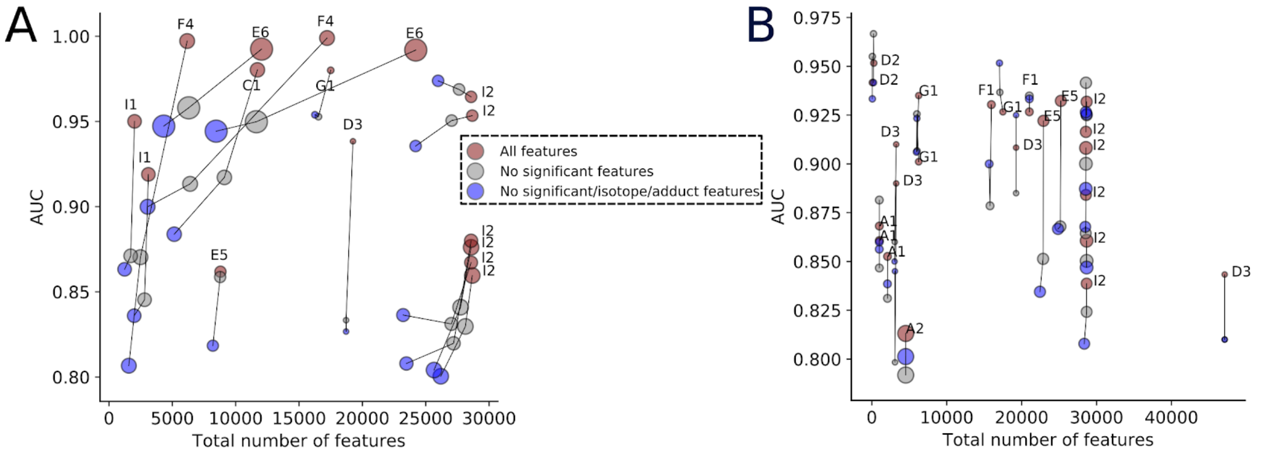

Figure S12. Studies with a larger number of participants tend to have more significant features (and hence adducts and isotopes); the total number of features plays a smaller role. (A) AUC relative to the total number of features used to train each model, in which the difference between the total number of features and the number of features remaining after removal of significant features, their adducts, and isotopes is > 500. (B) Same as in (A) but where the difference is < 500 (circle area proportional to study size for both).

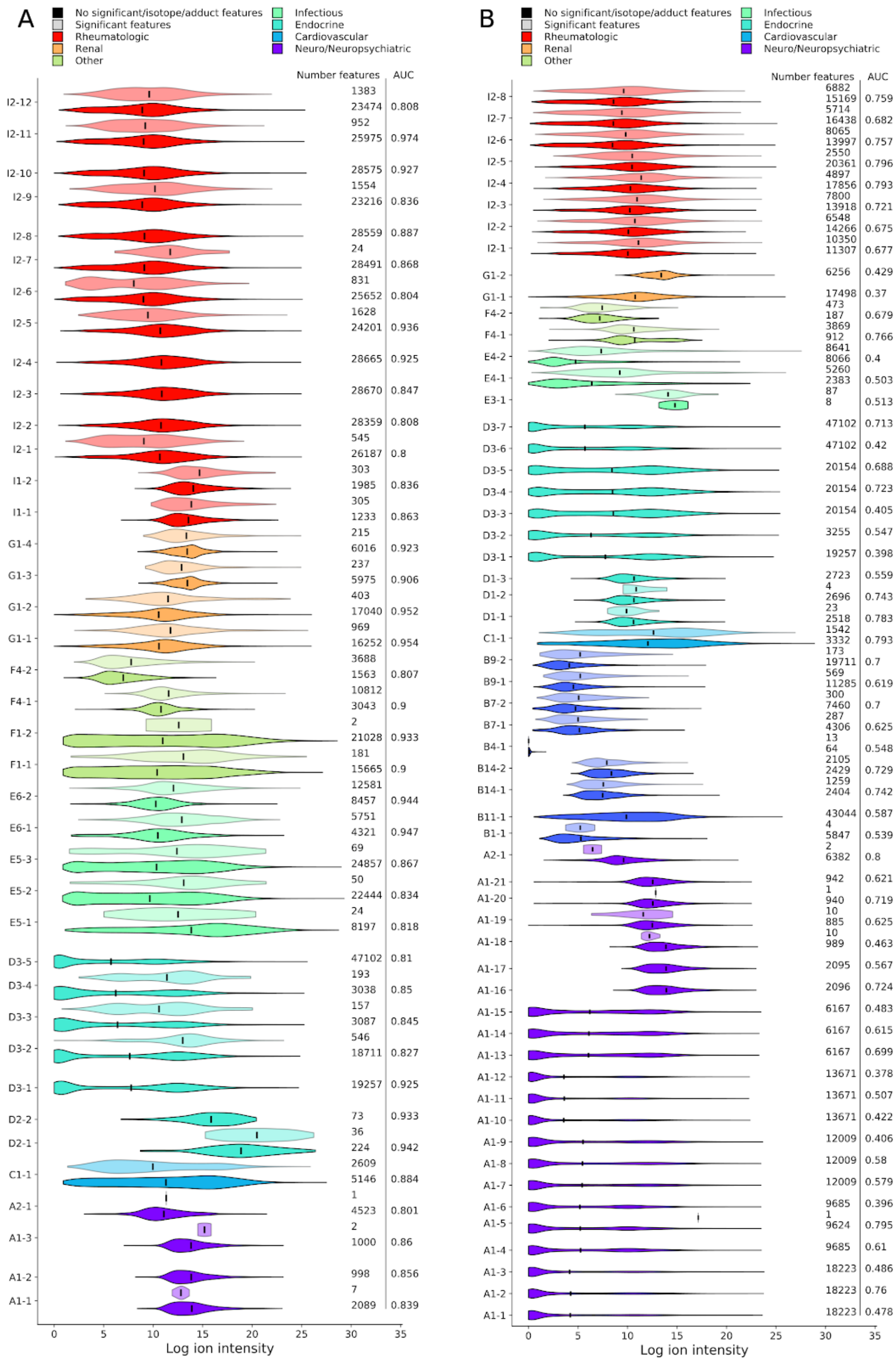

Figure S13. Intensities of non-significant, no adduct or isotope features often cover a similar range in intensities as the statistically significant metabolites. (A) data sets for which the AUC was greater than 0.8 and (B) Data sets for which the AUC was less than or equal to 0.8. Labels for data sets can be found in Table S2.

Table S2. Labels and data sets for Figure S22.

labels	data_set
A1-1	XCMS-Report-annotated-SingleClass.04jun12_CN_AD
A1-2	XCMS-Report-annotated-SingleClass.11jun12_CN_MCI
A1-3	XCMS-Report-annotated-SingleClass.11jun12_CN_AD
A2-1	IPO_aligned_MTBLS72_pos
C1-1	urine_onebatch_IPO_aligned_Feng_urine_all
D2-1	AN000929
D2-2	AN000930
D3-1	IPO_aligned_ST000045_2feb_pos_ND_IW
D3-2	IPO_aligned_ST000045_2feb_pos_II_IW
D3-3	IPO_aligned_ST000045_11feb_neg_ND_IW
D3-4	IPO_aligned_ST000045_11feb_neg_II_IW
D3-5	IPO_aligned_ST000045_17mar_neg_ND_IW
E5-1	IPO_aligned_MTBLS315_mzXML
E5-2	IPO_aligned_MTBLS315_n_mzML
E5-3	IPO_aligned_MTBLS315_p_mzML
E6-1	IPO_aligned_MTBLS354_neg
E6-2	IPO_aligned_MTBLS354_pos
F1-1	IPO_aligned_MTBLS266_neg
F1-2	IPO_aligned_MTBLS266_pos
F4-1	IPO_aligned_MTBLS364_hil_pos
F4-2	IPO_aligned_MTBLS364_lip_pos
G1-1	IPO_aligned_ST000329_pos_MCD_Control
G1-2	IPO_aligned_ST000329_pos_FSGS_Control
G1-3	IPO_aligned_ST000329_neg_MCD_Control
G1-4	IPO_aligned_ST000329_neg_FSGS_Control
I1-1	IPO_aligned_MTBLS408_neg
I1-2	IPO_aligned_MTBLS408_pos
I2-1	IPO_aligned_ST000763_untar_pos_Healthy_LowRisk
I2-2	IPO_aligned_ST000763_untar_pos_PAH_Normal Pressures
I2-3	IPO_aligned_ST000763_untar_pos_PAH_Borderline Pressures
I2-4	IPO_aligned_ST000763_untar_pos_Normal Pressures_Borderline Pressures
I2-5	IPO_aligned_ST000763_untar_pos_Normal Pressures_LowRisk
I2-6	IPO_aligned_ST000763_untar_neg_Healthy_LowRisk
I2-7	IPO_aligned_ST000763_untar_neg_PAH_Normal Pressures
I2-8	IPO_aligned_ST000763_untar_neg_PAH_Borderline Pressures
I2-9	IPO_aligned_ST000763_untar_neg_PAH_LowRisk
I2-10	IPO_aligned_ST000763_untar_neg_Normal Pressures_Borderline Pressures
I2-11	IPO_aligned_ST000763_untar_neg_Normal Pressures_LowRisk
I2-12	IPO_aligned_ST000763_untar_neg_Borderline Pressures_LowRisk

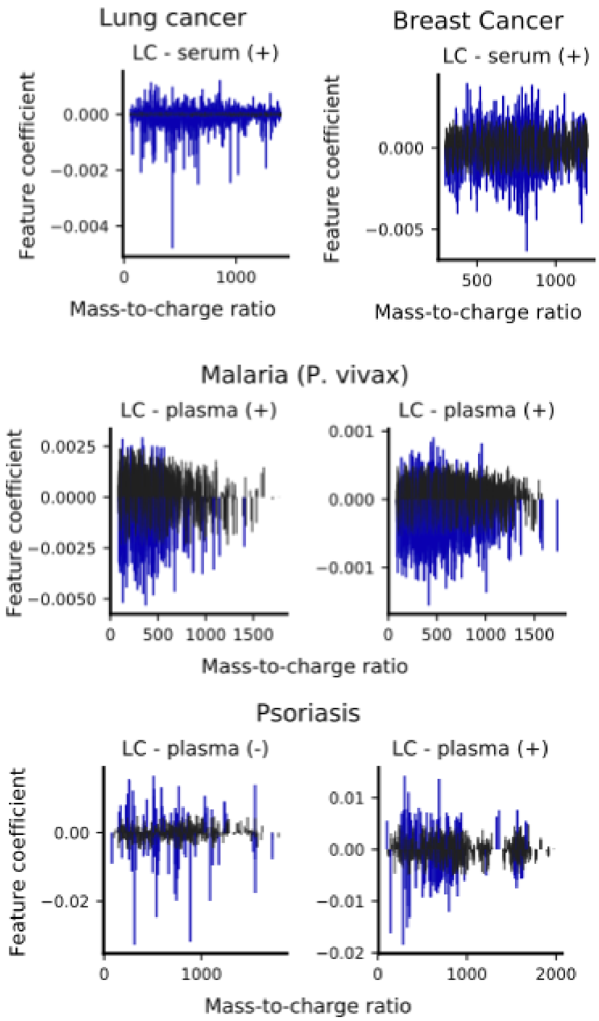

Figure S14. Mass-to-charge ratio versus model feature coefficient for individual data sets. Shown in blue are features that are in the top 5% highest feature coefficient in the model, while those in black are below this data set specific cutoff. Data sets (clockwise starting from upper left): Lung cancer - ST000388, Breast cancer - MTBLS92, Coronary Heart disease - Feng, Pneumonia - MTBLS354, Hepatocellular carcinoma - MTBLS19, Psoriasis - MTBLS408, Malaria - ST000578. Note: ST000608, ST000888 and ST000918 only had author feature tables of labels metabolites that could not be mapped to mz and thus not included.

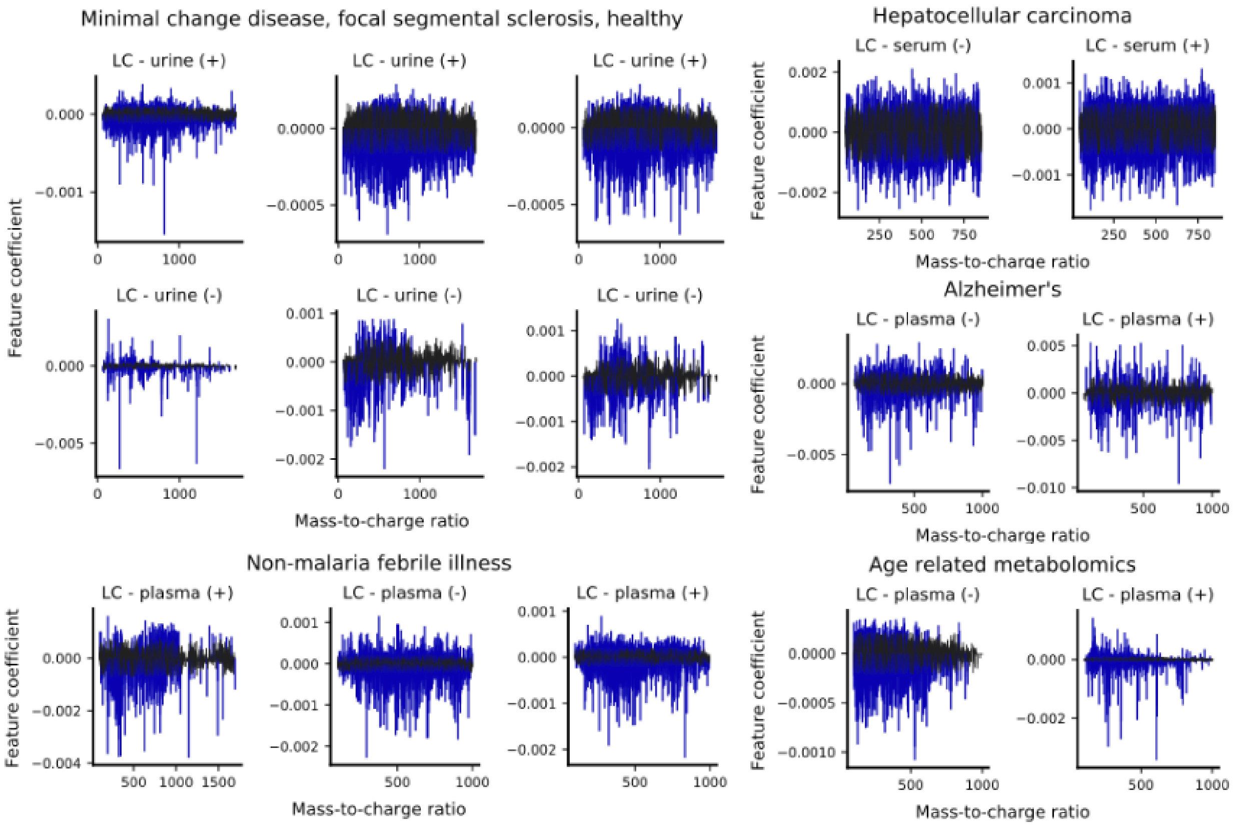

Figure S14 (continued). Mass-to-charge ratio versus model feature coefficient for individual data sets. Shown in blue are features that are in the top 5% highest feature coefficient in the model, while those in black are below this data set specific cutoff. Data sets (clockwise starting from upper left): Minimal change disease - ST000329, Hepatocellular carcinoma - MTBLS17, Alzheimer's - MTBLS72, Age related metabolomics - MTBLS266, Non-malaria febrile illness - MTBLS315,

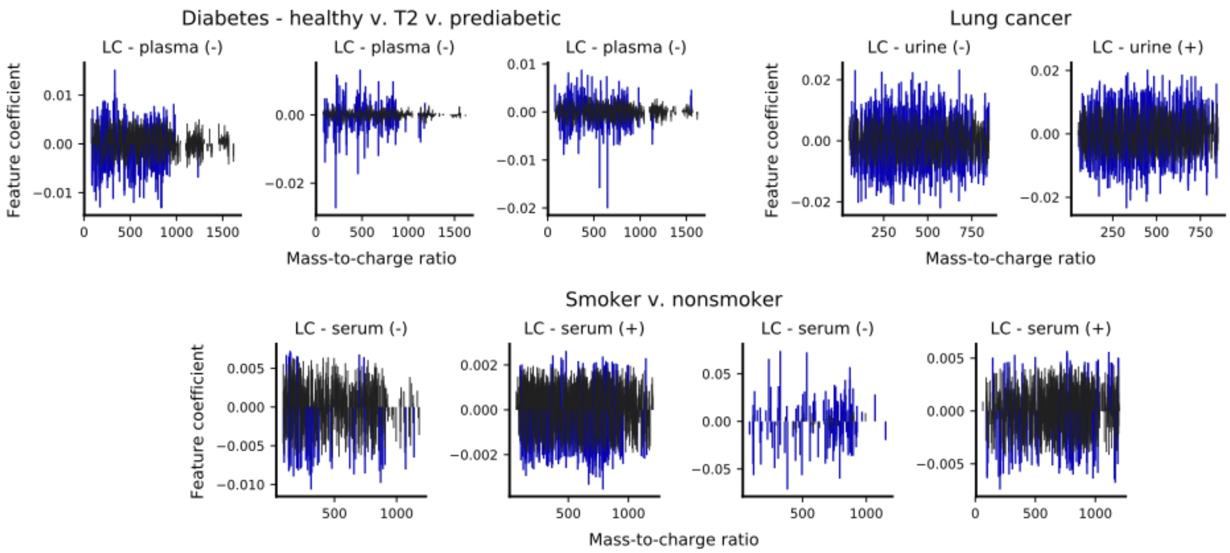

Figure S14 (continued). Mass-to-charge ratio versus model feature coefficient for individual data sets. Shown in blue are features that are in the top 5% highest feature coefficient in the model, while those in black are below this data set specific cutoff. Data sets (clockwise starting from upper left): Diabetes - MTBLS352, Lung cancer - MTBLS28, Smoker v. nonsmoker - MTBLS364.

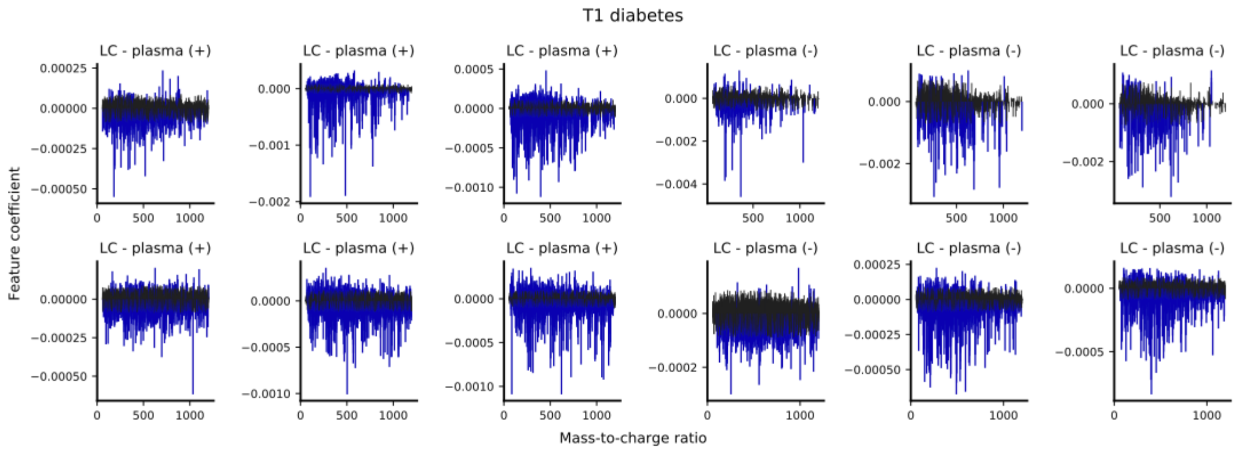

Figure S14 (continued). Mass-to-charge ratio versus model feature coefficient for individual data sets. Shown in blue are features that are in the top 5% highest feature coefficient in the model, while those in black are below this data set specific cutoff. Data sets: T1 Diabetes - ST000045.

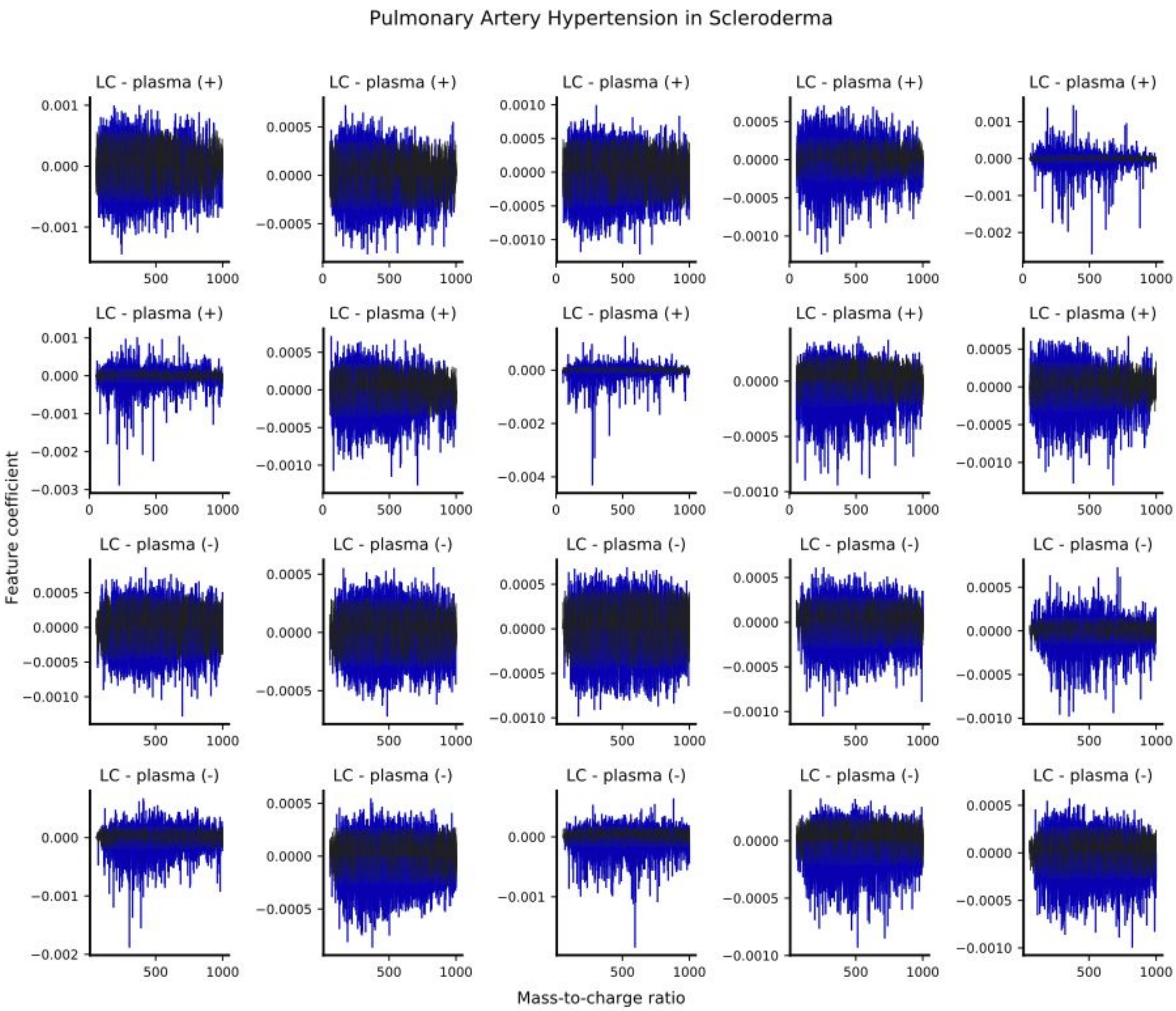

Figure S14 (continued). Mass-to-charge ratio versus model feature coefficient for individual data sets. Shown in blue are features that are in the top 5% highest feature coefficient in the model, while those in black are below this data set specific cutoff. Data set: Pulmonary artery hypertension - ST000763.

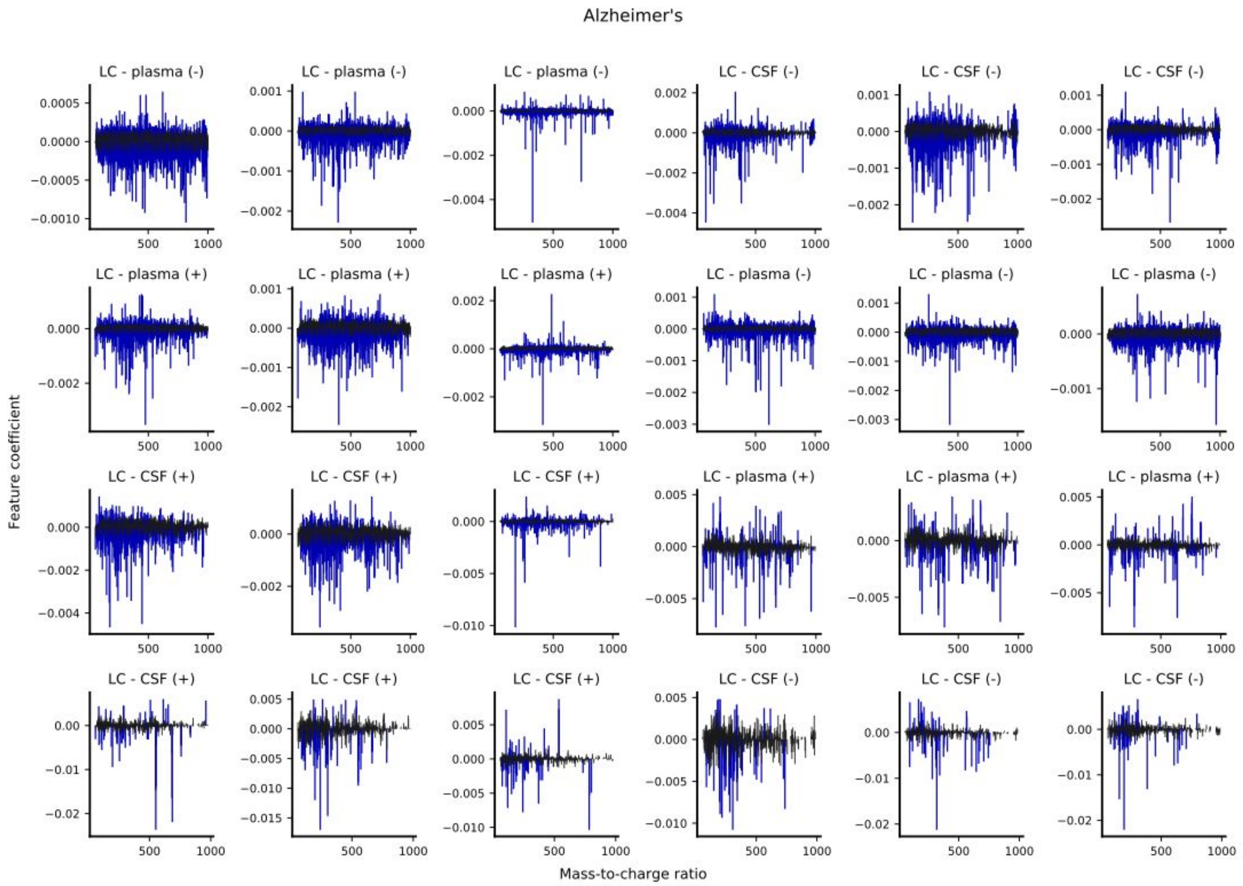

Figure S14 (continued). Mass-to-charge ratio versus model feature coefficient for individual data sets. Shown in blue are features that are in the top 5% highest feature coefficient in the model, while those in black are below this data set specific cutoff. Data set: Alzheimer's - ST000046.

References

1. Zeng, C. *et al.* Lipidomics profiling reveals the role of glycerophospholipid metabolism in psoriasis. *GigaScience* **6**, 1–11 (2017).
2. Hilvo, M. *et al.* Monounsaturated fatty acids in serum triacylglycerols are associated with response to neoadjuvant chemotherapy in breast cancer patients. *Int. J. Cancer* **134**, 1725–1733 (2014).
3. Xie, G. *et al.* Lowered circulating aspartate is a metabolic feature of human breast cancer. *Oncotarget* **6**, 33369–33381 (2015).
4. Cala, M. P. *et al.* Multiplatform plasma metabolic and lipid fingerprinting of breast cancer: A pilot control-case study in Colombian Hispanic women. *PLoS ONE* **13**, e0190958 (2018).
5. Zhu, J. *et al.* Colorectal Cancer Detection Using Targeted Serum Metabolic Profiling. *J. Proteome Res.* **13**, 4120–4130 (2014).
6. Xiao, J. F. *et al.* LC–MS Based Serum Metabolomics for Identification of Hepatocellular Carcinoma Biomarkers in Egyptian Cohort. *J. Proteome Res.* **11**, 5914–5923 (2012).
7. Ressom, H. W. *et al.* Utilization of metabolomics to identify serum biomarkers for hepatocellular carcinoma in patients with liver cirrhosis. *Anal. Chim. Acta* **743**, 90–100 (2012).
8. Ranjbar, M. R. N. *et al.* GC-MS Based Plasma Metabolomics for Identification of Candidate Biomarkers for Hepatocellular Carcinoma in Egyptian Cohort. *PLOS ONE* **10**, e0127299 (2015).
9. Poto, C. D. *et al.* Identification of race-associated metabolite biomarkers for hepatocellular carcinoma in patients with liver cirrhosis and hepatitis C virus infection. *PLOS ONE* **13**, e0192748 (2018).
10. Fahrmann, J. F. *et al.* Serum phosphatidylethanolamine levels distinguish benign from malignant solitary pulmonary nodules and represent a potential diagnostic biomarker for lung cancer. *Cancer Biomark.* **16**, 609–617 (2016).
11. Mathé, E. A. *et al.* Noninvasive Urinary Metabolomic Profiling Identifies Diagnostic and Prognostic Markers in Lung Cancer. *Cancer Res.* **74**, 3259–3270 (2014).
12. Miyamoto, S. *et al.* Systemic Metabolomic Changes in Blood Samples of Lung Cancer Patients Identified by Gas Chromatography Time-of-Flight Mass Spectrometry. *Metabolites* **5**, 192–210 (2015).
13. Fahrmann, J. F. *et al.* Investigation of Metabolomic Blood Biomarkers for Detection of Adenocarcinoma Lung Cancer. *Cancer Epidemiol. Prev. Biomark.* **24**, 1716–1723 (2015).
14. Wikoff, W. R. *et al.* Diacetylspermine Is a Novel Prediagnostic Serum Biomarker for Non–Small-Cell Lung Cancer and Has Additive Performance With Pro-Surfactant Protein B. *J. Clin. Oncol.* **33**, 3880–3886 (2015).
15. Feng, Q. *et al.* Integrated metabolomics and metagenomics analysis of plasma and urine identified microbial metabolites associated with coronary heart disease. *Sci. Rep.* **6**, 22525 (2016).
16. Schoeman, J. C. *et al.* Metabolic characterization of the natural progression of chronic hepatitis B. *Genome Med.* **8**, 64 (2016).
17. Molins, C. R. *et al.* Development of a metabolic biosignature for detection of early Lyme disease. *Clin. Infect. Dis. Off. Publ. Infect. Dis. Soc. Am.* **60**, 1767–1775 (2015).
18. Uppal, K. *et al.* Plasma metabolomics reveals membrane lipids, aspartate/asparagine and nucleotide metabolism pathway differences associated with chloroquine resistance in

Plasmodium vivax malaria. *PLOS ONE* **12**, e0182819 (2017).
19. Decuypere, S. *et al.* Towards Improving Point-of-Care Diagnosis of Non-malaria Febrile Illness: A Metabolomics Approach. *PLoS Negl. Trop. Dis.* **10**, e0004480 (2016).
20. To, K. K. W. *et al.* Lipid metabolites as potential diagnostic and prognostic biomarkers for acute community acquired pneumonia. *Diagn. Microbiol. Infect. Dis.* **85**, 249–254 (2016).
21. Näsström, E. *et al.* Diagnostic metabolite biomarkers of chronic typhoid carriage. *PLoS Negl. Trop. Dis.* **12**, e0006215 (2018).
22. Zhong, H. *et al.* Lipidomic profiling reveals distinct differences in plasma lipid composition in healthy, prediabetic, and type 2 diabetic individuals. *GigaScience* **6**, 1–12 (2017).
23. Fiehn, O. *et al.* Plasma metabolomic profiles reflective of glucose homeostasis in non-diabetic and type 2 diabetic obese African-American women. *PloS One* **5**, e15234 (2010).
24. Kyle, J. E. *et al.* Comparing identified and statistically significant lipids and polar metabolites in 15-year old serum and dried blood spot samples for longitudinal studies. *Rapid Commun. Mass Spectrom. RCM* **31**, 447–456 (2017).
25. Dutta, T. *et al.* Concordance of Changes in Metabolic Pathways Based on Plasma Metabolomics and Skeletal Muscle Transcriptomics in Type 1 Diabetes. *Diabetes* **61**, 1004–1016 (2012).
26. Trushina, E., Dutta, T., Persson, X.-M. T., Mielke, M. M. & Petersen, R. C. Identification of Altered Metabolic Pathways in Plasma and CSF in Mild Cognitive Impairment and Alzheimer's Disease Using Metabolomics. *PLOS ONE* **8**, e63644 (2013).
27. Mapstone, M. *et al.* Plasma phospholipids identify antecedent memory impairment in older adults. *Nat. Med.* **20**, 415 (2014).
28. Chaleckis, R., Murakami, I., Takada, J., Kondoh, H. & Yanagida, M. Individual variability in human blood metabolites identifies age-related differences. *Proc. Natl. Acad. Sci.* **113**, 4252–4259 (2016).
29. Naviaux, R. K. *et al.* Metabolic features of chronic fatigue syndrome. *Proc. Natl. Acad. Sci.* **113**, E5472–E5480 (2016).
30. Kind, T. *et al.* Interstitial Cystitis-Associated Urinary Metabolites Identified by Mass-Spectrometry Based Metabolomics Analysis. *Sci. Rep.* **6**, (2016).
31. Kaluarachchi, M. R., Boulangé, C. L., Garcia-Perez, I., Lindon, J. C. & Minet, E. F. Multiplatform serum metabolic phenotyping combined with pathway mapping to identify biochemical differences in smokers. *Bioanalysis* **8**, 2023–2043 (2016).
32. Titz, B. *et al.* Alterations in Serum Polyunsaturated Fatty Acids and Eicosanoids in Patients with Mild to Moderate Chronic Obstructive Pulmonary Disease (COPD). *Int. J. Mol. Sci.* **17**, 1583 (2016).
